# PERK induces resistance to cell death elicited by endoplasmic reticulum stress and chemotherapy

**DOI:** 10.1186/s12943-017-0657-0

**Published:** 2017-05-12

**Authors:** Iris C. Salaroglio, Elisa Panada, Enrico Moiso, Ilaria Buondonno, Paolo Provero, Menachem Rubinstein, Joanna Kopecka, Chiara Riganti

**Affiliations:** 1 Department of Oncology, University of Torino, via Santena 5/bis, 10126 Torino, Italy; 2 System Biology Ireland, University College Dublin, Dublin 4, Ireland; 3 Department of Molecular Biotechnology and Health Sciences, University of Torino, via Nizza 52, 10126 Torino, Italy; 4 Department of Molecular Genetics, The Weizmann Institute of Science, 234 Herzl Street, 7610001 Rehovot, Israel

**Keywords:** Eukaryotic translation initiation factor-2α kinase 3/protein kinase RNA-like endoplasmic reticulum kinase, Multidrug resistance related protein 1, Endoplasmic reticulum stress, Unfolded protein response, Chemoresistance

## Abstract

**Background:**

Nutrient deprivation, hypoxia, radiotherapy and chemotherapy induce endoplasmic reticulum (ER) stress, which activates the so-called unfolded protein response (UPR). Extensive and acute ER stress directs the UPR towards activation of death-triggering pathways. Cancer cells are selected to resist mild and prolonged ER stress by activating pro-survival UPR. We recently found that drug-resistant tumor cells are simultaneously resistant to ER stress-triggered cell death. It is not known if cancer cells adapted to ER stressing conditions acquire a chemoresistant phenotype.

**Methods:**

To investigate this issue, we generated human cancer cells clones with acquired resistance to ER stress from ER stress-sensitive and chemosensitive cells.

**Results:**

ER stress-resistant cells were cross-resistant to multiple chemotherapeutic drugs: such multidrug resistance (MDR) was due to the overexpression of the plasma-membrane transporter MDR related protein 1 (MRP1). Gene profiling analysis unveiled that cells with acquired resistance to ER stress and chemotherapy share higher expression of the UPR sensor protein kinase RNA-like endoplasmic reticulum kinase (PERK), which mediated the erythroid-derived 2-like 2 (Nrf2)-driven transcription of MRP1. Disrupting PERK/Nrf2 axis reversed at the same time resistance to ER stress and chemotherapy. The inducible silencing of *PERK* reduced tumor growth and restored chemosensitivity in resistant tumor xenografts.

**Conclusions:**

Our work demonstrates for the first time that the adaptation to ER stress in cancer cells produces a MDR phenotype. The PERK/Nrf2/MRP1 axis is responsible for the resistance to ER stress and chemotherapy, and may represent a good therapeutic target in aggressive and resistant tumors.

**Electronic supplementary material:**

The online version of this article (doi:10.1186/s12943-017-0657-0) contains supplementary material, which is available to authorized users.

## Background

Cancer cells often face conditions of nutrient deprivation, hypoxia, alterations in glycosylation status and calcium flux [1, 2], leading to the accumulation of unfolded or misfolded proteins in the endoplasmic reticulum (ER) lumen. These conditions activate the so-called unfolded protein response (UPR; [3, 4]). In the early phase, UPR mediates the adaptation to stress by modifying the transcriptional and translational programs responsible for protein folding, and/or by promoting ER-associated degradation (ERAD) pathways to remove misfolded proteins. If the attempt to adapt to ER stress fails, the UPR activates cell death programs to eliminate the damaged cells [5, 6]. Recently, we demonstrated that cancer cells with constitutive or acquired resistance to chemotherapy are also resistant to ER stress-triggered cell death. This resistance was due to ubiquitination and subsequent degradation of the ER stress-activated transcription factor CAAT/enhancer-β liver-enriched inhibitory protein (C/EBP-β LIP). Consequently, the pro-apoptotic axis C/EBP homologous protein (CHOP)/caspase 3 was down-regulated in the resistant cells. Moreover, LIP elimination in the chemoresistant cells up-regulates the transcription of P-glycoprotein (Pgp) [7]. Pgp prevents the accumulation of chemotherapeutic drugs and targeted therapy-agents, determining multidrug resistance (MDR) [8]. Since many chemotherapeutic drugs – such as anthracyclines [9], cisplatin [10], oxaliplatin [9], 5-fluorouracil [11] and paclitaxel [12] – induce a massive ER-stress mediated cell death, the lack of ER stress-dependent apoptotic response coupled with the increased drug efflux strongly reduces the efficacy of these drugs in MDR cells.

Three sensors of ER stress - activating transcription factor 6 (ATF6), inositol-requiring enzyme 1 (IRE1) and protein kinase RNA-like endoplasmic reticulum kinase/ eukaryotic translation initiation factor-2α kinase 3 (PERK) - initiate the UPR in cancer cells [3, 13]. Activated PERK phosphorylates eukaryotic translation initiation factor-2α (EIF2α) and nuclear factor erythroid-derived 2-like 2 (Nrf2), thereby attenuating protein translation and inducing genes controlling redox homeostasis [13, 14]. The activation of PERK may determine resistance to ER stress-induced cell death in a EIF2α-dependent manner [6, 13] and resistance to chemotherapy-induced cell death in a Nrf2-dependent manner [15–18]. By binding antioxidant response elements (AREs) in promoter regions, Nrf2 up-regulates antioxidant genes, metal-binding proteins, stress response proteins, drug-metabolizing enzyme and drug efflux transporters such as MDR-related protein 1 (MRP1) [16, 19].

While our previous findings suggest that cells with acquired resistance to chemotherapy are also resistant to ER stress-dependent cell death, it is not known whether chemosensitive cancer cells adapted to ER stress acquire resistance to chemotherapy as well. To address this issue, we exposed ER stress-sensitive/chemosensitive cancer cells to 3 different ER stress inducers developing the corresponding ER stress-resistant clones. We demonstrated that the activation of PERK/Nrf2/MRP1 axis determines the resistance to ER stress and also resistance to chemotherapy. Inhibiting this axis is an effective anti-proliferative and chemosensitizing strategy.

## Methods

### Chemicals and supplies

Cell culture plasticware were obtained from Falcon (Becton Dickinson, Franklin Lakes, NJ). Gel electrophoresis reagents were obtained from Bio-Rad Laboratories (Hercules, CA). The protein content of cell lysates was measured with the BCA kit from Sigma Chemicals Co. (St. Louis, MO) using bovine serum albumin as a standard. Unless specified otherwise, all other reagents were purchased from Sigma Chemical Co.

### Cells

Human chemosensitive colon cancer HT29 cells (ATCC, Manassas, VA) were cultured in RPMI-1640 medium supplemented with 10% fetal bovine serum (FBS, Invitrogen Life Technologies, Carlsbad, CA) and 100 U/ml penicillin, 100 μg/ml streptomycin. HT29/MDR cells, a subpopulation of HT29 cells displaying resistance to chemotherapy and ER stress inducers (Additional file 1: Table S1), were obtained as reported [7]. ER stress-resistant subpopulations, termed HT29/Tg, HT29/Tun and HT29/Bfa, were created by stepwise selection of parental HT29 cells in RPMI-1640 medium containing increasing concentrations of thapsigargin, tunicamycin and brefeldin A, respectively. Resistant subclones were then maintained at a concentration of each ER stress inducer that allowed > 95% cell viability (500 nM thapsigargin, 250 nM tunicamycin and 250 nM brefeldin A). ER stress-resistant clones were similarly derived from human chemosensitive breast cancer MCF7 cells and human chemosensitive osteosarcoma U-2OS cells (ATCC). MCF7/Tun and U-2OS/Tun were maintained in DMEM/F12 and IMDM medium (Invitrogen Life Technologies), supplemented with 10% FBS, 100 U/ml penicillin, 100 μg/ml streptomycin, containing 500 nM tunicamycin. When cultured in 3D-systems, 1 × 10^5^ cells were seeded in 96-well plate coated with Biomimesys™ matrix (Celenys, Rouen, France). After 7 days, 3D-cultures were treated and analyzed by contrast phase Leica DC100 microscope (Leica Microsystems GmbH, Wetzlar, Germany; 10X ocular lens, 4X objective). Cell lines were authenticated by microsatellite analysis using the PowerPlex kit (Promega Corporation, Madison, WI; last authentication: September 2016).

### Measurement of cell necrosis and cell viability

Cells were incubated for 24 h (for the High Mobility Group Protein 1, HMGB1, assay) or 48 h (for Neutral red and crystal violet staining) in fresh medium, or in medium containing thapsigargin (10 μM), tunicamycin (1 μM), brefeldin A (1 μM), or the chemotherapeutic drugs oxaliplatin or cisplatin (10 μM), 5-fluorouracil (5 μM), doxorubicin (5 μM). Acute cell toxicity was measured by evaluating the release of HMGB1 in the cell culture supernatant, using the High Mobility Group Protein 1 ELISA kit (Cloud-Clone Corp., Houston, Texas). Results were expressed in pg/mg total cellular protein, employing a pre-made titration curve. Cell viability was measured using Neutral red staining assay [7]. The viability of untreated cells was considered 100%; the results were expressed as percentage of viable cells in each experimental condition versus untreated cells. IC_50_ was calculated by incubating cells with increasing concentrations of the drugs (from 10^−10^ to 10^- 3^ M), then staining cells with Neutral red. To evaluate morphology, cells were stained with 5% w/v crystal violet solution in 66% v/v methanol, washed and analyzed under bright field microscope (10X objective; 10X ocular lens).

### Immunoblotting

Cells were rinsed with ice-cold lysis RIPA buffer (50 mM Tris, 10 mM EDTA, 1% v/v Triton-X100; pH 7.5), supplemented with the protease inhibitor cocktail set III (80 μM aprotinin, 5 mM bestatin, 1.5 mM leupeptin, 1 mM pepstatin; Calbiochem, San Diego, CA), 2 mM phenylmethylsulfonyl fluoride and 1 mM Na_3_VO_4_. The cells were then sonicated (10 bursts of 10 s, 4 °C, 100 W, using a Labsonic sonicator, Hielscher, Teltow, Germany) and centrifuged at 13,000 × g for 10 min at 4 °C. 20 μg protein extracts were subjected to 4-20% gradient SDS-PAGE and probed with the following antibodies: anti-Pgp (1:500; Calbiochem); anti-MRP1 (1:500; Abcam, Cambridge, UK); anti-MRP2 (1:250; Abcam); anti-MRP3 (1:500; Santa Cruz Biotechnology Inc., Santa Cruz, CA); anti-MRP4 (1:250; Santa Cruz Biotechnology Inc.); anti-MRP5 (1:500; Abcam); anti-BCRP (1:1000; Santa Cruz Biotechnology Inc.), anti-PERK (1:500; Santa Cruz Biotechnology Inc.), anti-IRE1 (1:500; Thermo Scientific Inc., Rockford, IL), anti-ATF6 (1:500; Santa Cruz Biotechnology Inc.), anti-eIF2α (1:1000; Abcam), anti-phospho(Ser51)eIF2α (1:500; Abcam), anti-β-tubulin (1:1000; Santa Cruz Biotechnology Inc.). The membranes were then incubated with peroxidase-conjugated secondary antibody (1:3000; Bio-Rad Laboratories) and washed with Tris-buffered saline-Tween 0.1% v/v solutions. Protein bands were detected by enhanced chemiluminescence (Bio-Rad Laboratories). Nuclear extracts were prepared using the Nuclear Extract kit (Active Motif, La Hulpe, Belgium). Nuclear proteins were separated by SDS-PAGE and probed with anti-Nrf2 (1:500; Abcam) or anti-TATA box binding protein antibodies (TBP, 1:500; Santa Cruz Biotechnology Inc.).

### Quantitative Real Time-PCR (qRT-PCR) and PCR expression arrays

Total RNA was extracted and reverse-transcribed using the iScript™ cDNA Synthesis Kit (Bio-Rad Laboratories). qRT-PCR was performed using IQ™ SYBR Green Supermix (Bio-Rad Laboratories). The same cDNA preparation was used for measuring genes of interest and the housekeeping gene *S14*. The primer sequences, designed with qPrimerDepot software (https://primerdepot.nci.nih.gov/), are reported in the Additional File 2. Relative gene expression levels were calculated using Gene Expression Quantitation software (Bio-Rad Laboratories). PCR arrays were generated using 1 μg cDNA and Human Unfolded Protein Response Plus RT^2^ Profiler*™* PCR Array (Bio-Rad Laboratories) according to the manufacturer’s instructions. Data analysis was performed using the PrimePCR™ Analysis Software (Bio-Rad Laboratories).

### Flow cytometry

Cells (1 × 10^6^) were rinsed and fixed with 2% w/v paraformaldehyde for 2 min, permeabilized using 0.1% v/v Triton-X100 for 2 min on ice, washed three times with PBS and stained with an anti-MRP1 antibody (1:250, Abcam) for 1 h on ice. The cells were then incubated with an AlexaFluor 488-conjugated secondary antibody (1:100, Millipore, Billerica, MA) for 30 min and washed again. Samples were analyzed with a FACS-Calibur flow cytometer (Becton Dickinson). For each analysis 10000 events were collected. Control experiments included incubation with non immune isotype antibody followed by the secondary antibody. The results were expressed as mean fluorescence value of MRP1 expression, calculated with the Cell Quest software (Becton Dickinson).

### Intracellular doxorubicin accumulation

Doxorubicin content was measured by fluorimetry as detailed elsewhere [20]. The results were expressed as nmol doxorubicin/mg cell proteins, according to a pre-formed titration curve.

### Chromatin immunoprecipitation (ChIP)

ChIP experiments were performed for determining binding of Nrf2 to the ARE1 site of the *MRP1* promoter [21]. The PCR primers used were: 5’-CGGCTCGAGTTATCATGTCTCCAGGCTTCA-3’; 5’-CGGAAGCTTGCCGGTGGCGCGGG-3’.

### *PERK* silencing

Cells (2 × 10^6^ in 0.25 mL FBS/antibiotic-free medium) were transduced with 6 × 10^5^ lentiviral particles (Thermo Scientific Open Biosystems, Waltham, MA). 6 h after the transfection, 0.25 mL complete medium was added. Medium was fully replaced 24 h after the transfection. Transfection efficiency was checked by evaluating the percentage of green fluorescent protein (GFP)-positive cells by fluorescence microscopy, 48 h after the transfection: in each experiment, GFP-positive cells were ≥90%. Stably transduced clones were selected by culturing cells in medium containing 2 μg/mL puromycin, for 3 weeks. *PERK* shRNA was induced by adding 1 μg/mL doxycycline to the culture medium for 72 h. To verify the silencing efficacy, cells were lysed and PERK was visualized by immunoblotting, as described above.

### In vivo tumor growth

HT29 cells or HT29/MDR cells (1 × 10^6^) transduced with the inducible silencing vector for *PERK*, were re-suspended in 100 μL culture medium, mixed with 100 μL Matrigel and injected s.c. into 6–8 weeks old NOD SCID BALB/c female mice (weight: 20.82 ± 2.34 g), housed under 12 h light/dark cycle, with food and drinking provided ad libitum. Tumor growth was measured daily by caliper, and was calculated according to the equation (LxW^2^)/2, where L = tumor length and W = tumor width. When tumor reached the volume of 100 mm^3^, mice were randomized and treated on day 3, 9, 15 as it follows: 1) Ctrl group was treated with 200 μL saline solution i.v.; 2) oxaliplatin (oPt) group was treated with 5 mg/Kg oPt i.p. Intratumor *PERK* silencing was activated by doxycycline (2 mg/mL) in the drinking water. Animals were euthanized at day 21. Tumors were resected, photographed and fixed in 4% v/v paraformaldehyde. The paraffin sections were stained with hematoxylin/eosin or immunostained for PERK (1:50), MRP1 (1:50), cleaved caspase 3 (Asp175, 1:50; Cell Signaling Technology Inc., Danvers, MA), followed by a peroxidase-conjugated secondary antibody (1:100, Dako, Glostrup, Denmark). Sections were examined with a Leica DC100 microscope (Leica Microsystems GmbH, Wetzlar, Germany; 10X ocular lens, 63X objective).

### Cell migration

In vitro migration was evaluated by the scratch wound healing assay over a period of 24 h, as reported [22]. Results were expressed as μm/h, by performing ≥ 100 measurement per each condition.

### Statistical analysis

All data in text and figures are provided as means ± SD. The results were analyzed by a one-way Analysis of Variance (ANOVA). *p* < 0.05 was considered significant.

Gene expression profiles and clinical data were obtained from The Cancer Genome Atlas (TCGA; https://cancergenome.nih.gov/) and analysed using R (https://www.R-project.org). Survival association analyses were performed using Cox's proportional hazard model in univariate setting and Kaplan-Meier method, applying false discovery rate (FDR) and Bonferroni correction for multiple testing.

## Results

### Cells adapted to ER stress acquire resistance to chemotherapy

**Fig. 1:**
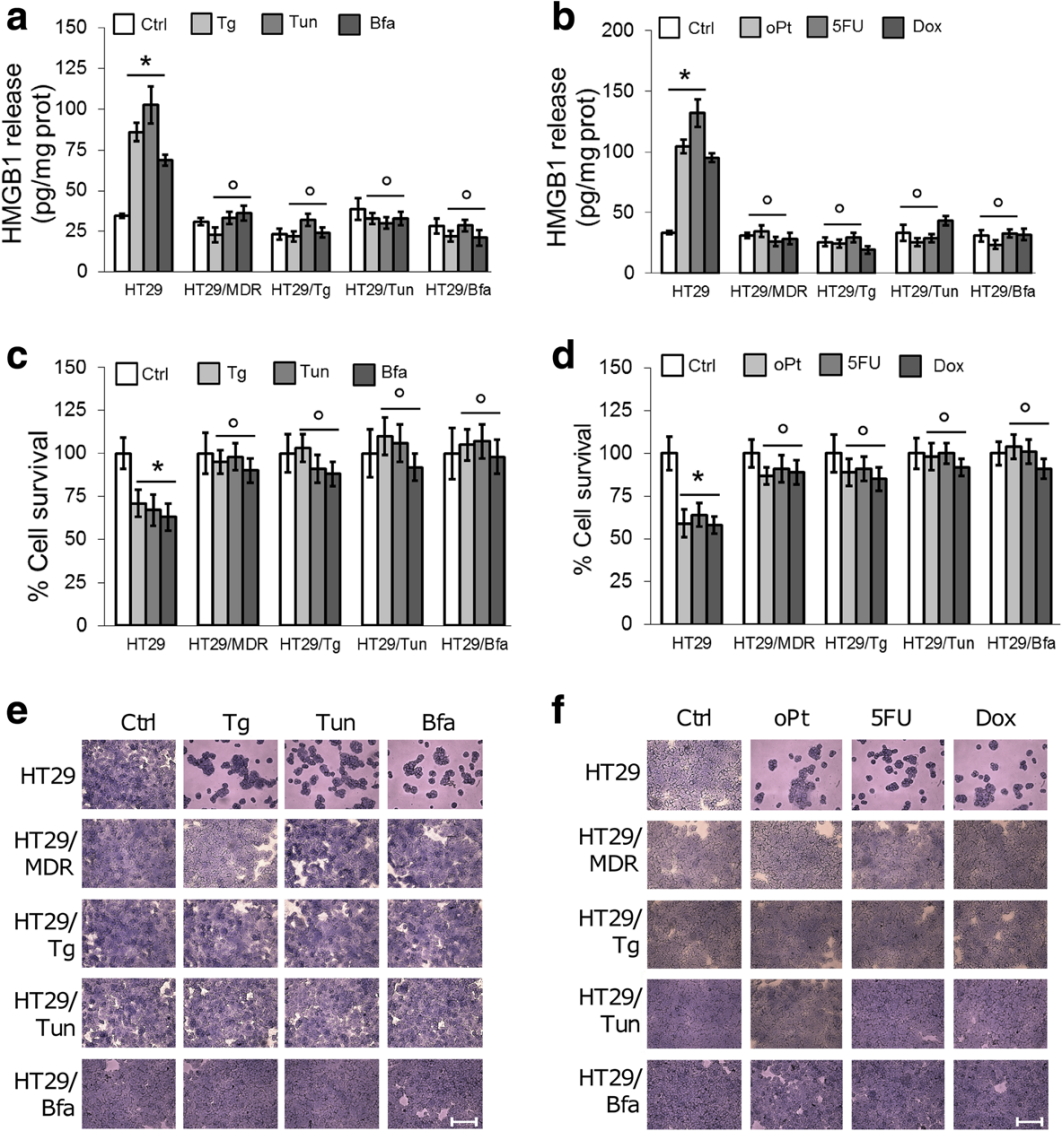
Drug resistance of human colon cancer cells adapted to ER stress. **a**, **b**. Release of the necrosis marker HMGB1 to culture media of the indicated cells (human chemosensitive HT29 cells, chemoresistant HT29/MDR cells, ER stress-resistant clones HT29/Tg, HT29/Tun, HT29/Bfa), following incubation in fresh medium (Ctrl), or in media containing: thapsigargin (Tg), tunicamycin (Tun), brefeldin A (Bfa), oxaliplatin (oPt), 5-fluorouracil (5FU), doxorubicin (Dox), as indicated in Methods. Data are mean ± SD. (*n* = 3). *p < 0.001 for treated cells vs. Ctrl HT29 cells. °*p* < 0.001 for HT29/MDR, HT29/Tg, HT29/Tun/HT29/Bfa cells vs. the corresponding condition in HT29 cells. **c**, **d**. Viability of cells measured by Neutral red staining. Data are mean ± SD (*n* = 4). *p < 0.02 for treated cells vs. Ctrl HT29 cells; °*p* < 0.05 for HT29/MDR, HT29/Tg, HT29/Tun/HT29/Bfa cells vs. the corresponding condition in HT29 cells. **e**, **f**. Crystal violet staining of cells grown in 96-well plates and treated as in **a** and **b**. The images are representatives of at least 5 microscopic fields showing similar cell density. Bars = 500 μM

Chemosensitive human colon cancer HT29 and its chemoresistant clone HT29/MDR were incubated with the ER stress inducers thapsigargin, tunicamycin or brefeldin A, or with the chemotherapeutic agents oxaliplatin (a substrate of MRP1 and MRP4), 5-fluorouracil (a substrate of MRP1, MRP3, MRP4 and MRP5) or doxorubicin (a substrate of Pgp, MRP1, MRP2, MRP3 and BCRP), at concentrations that were cytotoxic in chemosensitive cells but not in chemoresistant ones ([7]; Additional file 1). Cytotoxicity was characterized by release of HMGB1 to the culture media and by reduced cell viability (Fig. 1a-f). To verify whether cells resistant to ER stress were also cross-resistant to chemotherapy, we generated the ER stress resistant clones HT29/Tg, HT29/Tun, HT29/Bfa from the ER stress-sensitive/chemosensitive HT29 cells. In these clones neither ER stress inducers nor chemotherapeutic agents increased the release of HMGB1 (Fig. 1a, b) or reduced cell viability (Fig. 1c-f). Resistance to oxaliplatin, chosen as a paradigmatic first-line treatment in colon cancer, was preserved in 3D-cultures of HT29/MDR and HT29/Tun cells (Additional file 3). Overall, these data suggest that the acquisition of resistance to ER stress is associated to the acquisition of resistance to chemotherapy.

### ER stress- and chemotherapy-resistant cells up-regulate MRP1

**Fig. 2:**
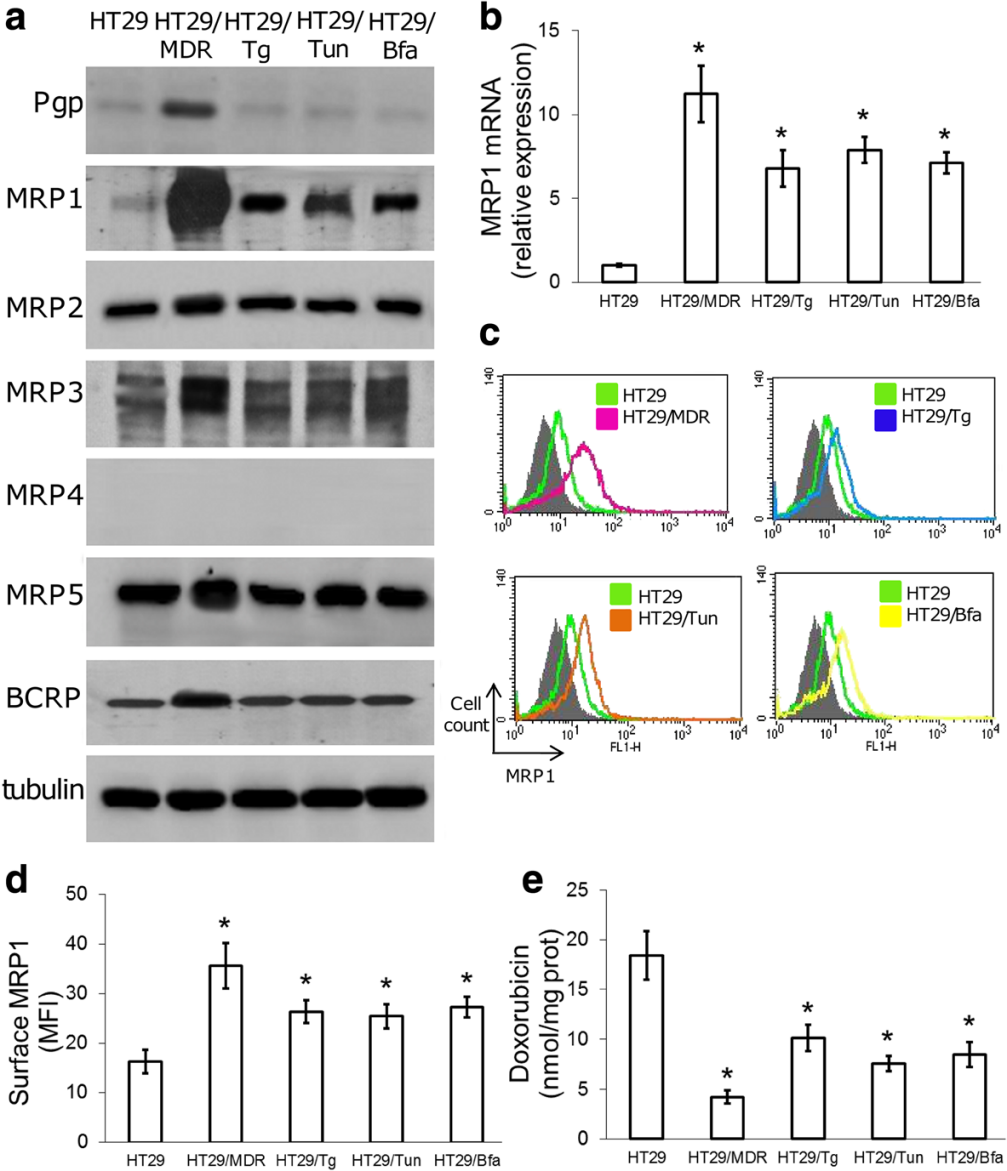
Expression of MRP1 in cells resistant to chemotherapy and to ER stress. **a**. Immunoblots of the indicated proteins in extracts of untreated cells. β-tubulin was used as a loading control. The figure is representative of 3 experiments with similar results. **b**. *MRP1* mRNA level as measured by qRT-PCR. Data are mean ± SD (*n* = 4). *p < 0.001 vs. HT29 cells. **c.** Representative flow cytometry histograms of MRP1 protein. Grey peaks: non immune isotypic antibody. **d**. Cell surface MRP1 was determined by flow cytometry. Data are mean fluorescence intensity (MFI) ± SD (*n* = 3). *p < 0.02 vs. HT29 cells. **e**. Intracellular doxorubicin content, an index of MRP1 activity, measured by fluorimetry. Data are mean ± SD (*n* = 3). *p < 0.001 vs. HT29 cells

Chemoresistance is often mediated by increased expression of ABC transporters [8]. Therefore, we analyzed their expression level in chemosensitive/ER stress-sensitive HT29 cells and in the HT29/Tg, HT29/Tun and HT29/Bfa clones. HT29/MDR were used as control of chemoresistant/ER-stress resistant cells overexpressing Pgp, MRP1, MRP2, MRP3, MRP5 and BCRP. Compared to HT29 cells, all the ER stress-resistant clones showed higher expression of MRP1 at the protein (Fig. 2a) and mRNA (Fig. 2b) levels, associated with a higher amount of MRP1 on the cell surface (Fig. 2c-d). In line with this trend, the intracellular accumulation of doxorubicin, which is inversely related to MRP1 activity, was lower in the ER-stress resistant clones, as well as in HT29/MDR cells (Fig. 2e).

To verify whether the cross resistance to ER stress inducers and to chemotherapeutic drugs was limited to HT29 cells or not, we generated two other ER stress-resistant clones, MCF7/Tun and U-2OS/Tun, from ER stress-sensitive/chemosensitive breast cancer MCF7 cells and osteosarcoma U-2OS cells. As for HT29 subclones, MCF7/Tun and U-2OS/Tun cells were resistant to both ER stress inducers and to chemotherapeutic drugs. These cells exhibited higher expression of MRP1 protein and mRNA, higher amount of MRP1 on cell surface, and lower retention of doxorubicin (Additional files 4 and 5). These data suggested that up-regulation of MRP1 is associated with the dual resistance to ER stress and to chemotherapy and is exhibited by a variety of cancer cells of different histological origin.

### The PERK/Nrf2 axis up-regulates MRP1 and controls the resistance to ER stress and to chemotherapy

To investigate whether there is a common gene signature between chemoresistant cells and ER stress-resistant cells, we compared the expression of 83 genes involved in UPR in HT29/Tun, HT29/MDR and HT29 cells.

**Fig. 3:**
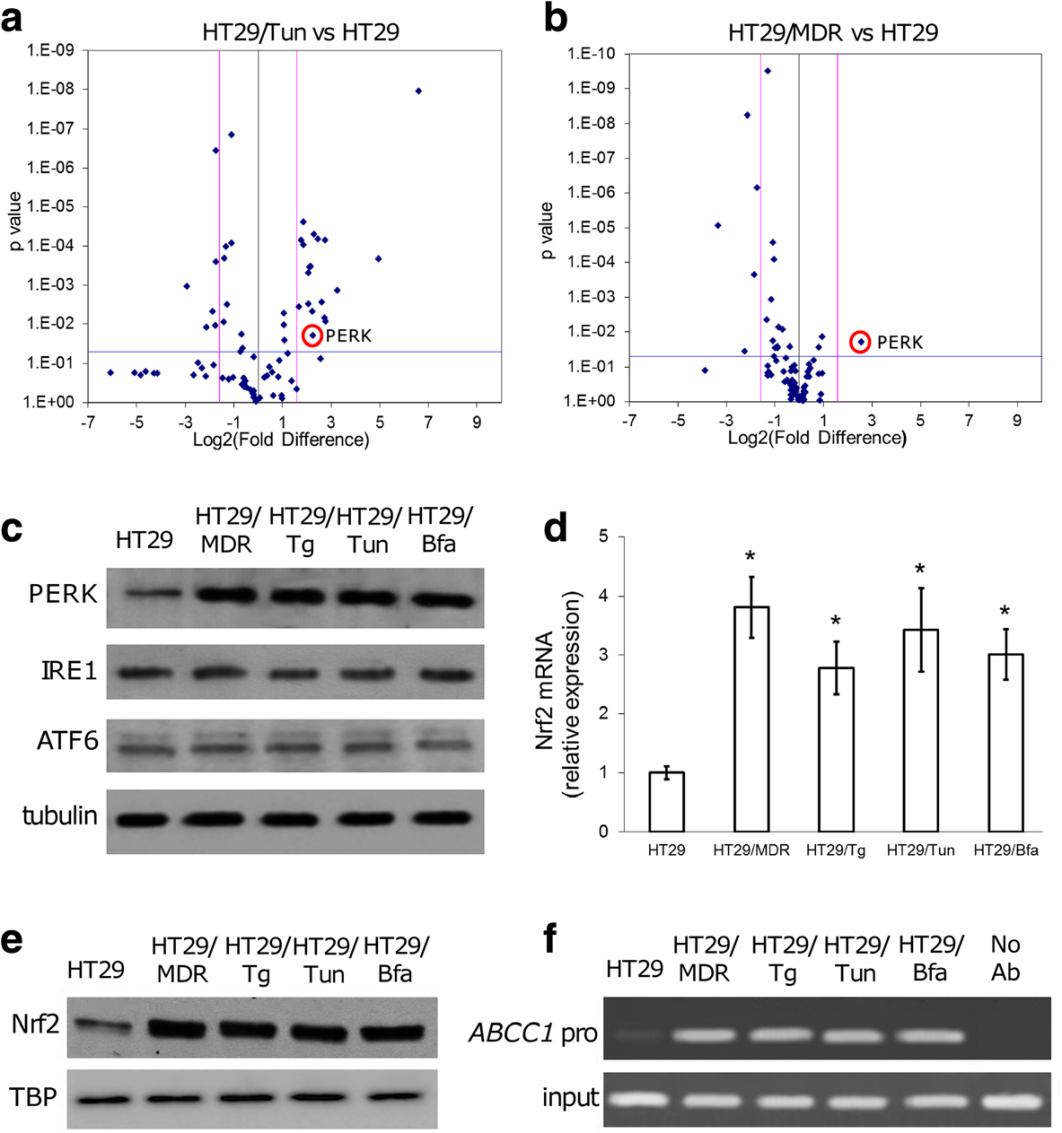
PERK expression in cells resistant to chemotherapy and to ER stress. **a**, **b**. Relative expression of 83 UPR genes in untreated HT29/Tun vs. HT29 cells (**a**), and in untreated HT29/MDR vs. HT29 cells (**b**). The Volcano plots are representative of 4 independent experiments. The spots corresponding to PERK are encircled. **c**. Immunoblots of the indicated proteins in extracts of untreated cells. β-tubulin was used as a loading control. The figure is representative of 3 experiments with similar results. **d**. *Nrf2* mRNA levels in extracts of untreated cells. Data are mean ± SD (*n* = 4). *p < 0.005 vs. HT29 cells. **e**. Immunoblot of Nrf2 in nuclear extracts of the indicated cells. TATA-box binding protein (TBP) served as a loading control. The figure is representative of 3 experiments with similar results. **f**. Binding of Nrf2 to the *ABCC1/MRP1* promoter (*ABCC1* pro) as measured by ChIP. The figure is representative of 3 experiments with similar results. Amplification of *ABCC1* promoter from genomic DNA (input) was used as control of equal DNA loading. No Ab: HT29/MDR DNA fragments were immunoprecipitated without the anti-Nrf2 antibody and used as a negative control

As expected, several genes involved in protein synthesis, ER quality control and ERAD were significantly up-regulated in HT29/Tun cells (Fig. 3a; Additional file 6). By contrast, only *PERK* was significantly increased in HT29/MDR cells (Fig. 3b; Additional file 6). Interestingly, the fold-increase of *PERK* mRNA in HT29/Tun and HT29/MDR cells was very similar (Fig. 3a, b; Additional file 6) and was associated with increased PERK protein levels (Fig. 3c). No appreciable change in the expression of the other ER stress sensors IRE1 and ATF6 was observed (Fig. 3c).

In line with previous findings [13, 14], the highly PERK-expressing HT29/MDR, HT29/Tg, HT29/Tun and HT29/Bfa cells had higher mRNA levels of the PERK-target/redox-sensitive factor *Nrf2* (Fig. 3d). Nrf2 protein was also more translocated in the nucleus (Fig. 3e) and it was bound to the *ABCC1/MRP1* promoter (Fig. 3e). Overall, these data suggest that the increase of MRP1 expression in cells resistant to ER stress and to chemotherapy is associated to up-regulation of PERK and Nrf2.

### Targeting the PERK/Nrf2/MRP1 axis abrogates the dual resistance to ER stress and chemotherapy

**Fig. 4:**
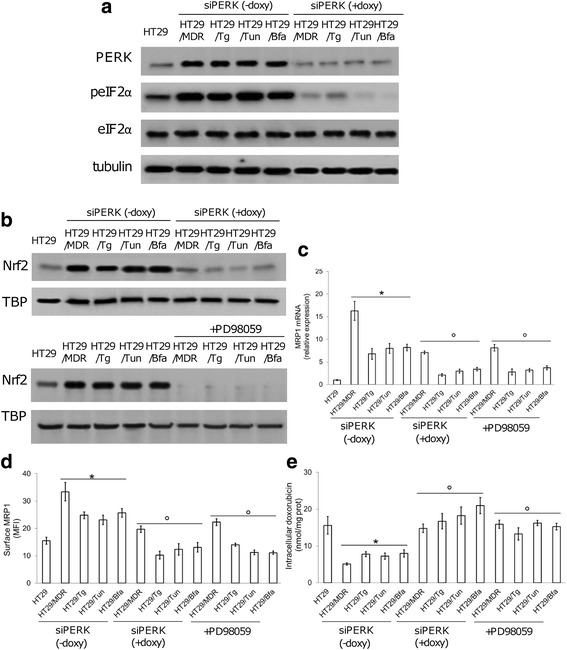
Role of the PERK/Nrf2 axis in MRP1 expression and activity. Human chemoresistant colon cancer HT29/MDR cells and ER stress-resistant clones (HT29/Tg, HT29/Tun, HT29/Bfa) were stably and inducibly silenced for *PERK* (siPERK). Silencing was induced by doxycycline (doxy, 1 μg/mL, 72 h). HT29 cells were used as control of chemosensitive/ER stress-sensitive cells. **a**. Immunoblots of protein extracts from the indicated cells stably and inducibly silenced for *PERK* (siPERK). β-tubulin was used as a loading control. The figure is representative of 3 experiments with similar results. **b**. Immunoblots of Nrf2 in nuclear extracts of cells stably and inducibly silenced for *PERK*, or treated with PD98059 (10 μM, 72 h), which prevents Nrf2 nuclear translocation. TBP was used as a loading control. The figure is a representative of 3 experiments with similar results. **c**. Total *MRP1* mRNA in extracts of the indicated cells. Data are mean ± SD (*n* = 4). *p < 0.001 for siPERK-treated cells vs. untreated HT29 cells; °*p* < 0.001 for siPERK + doxy-treated cells/PD98059-treated cells, vs. the corresponding condition in siPERK – doxy cells. **d**. Cell surface MRP1 as measured by flow cytometry. Data are mean fluorescence intensity (MFI) ± SD. (*n* = 3). *p < 0.002 for siPERK(− doxy)-treated cells vs. untreated HT29 cells; °*p* < 0.005 for siPERK(+ doxy)-treated cells and PD98059-treated cells vs. the corresponding cell type in siPERK(−doxy) cells. **e**. Doxorubicin uptake in cells treated as above and measured by fluorimetry. Data are mean ± SD (*n* = 3). *p < 0.001 for siPERK(− doxy)-cells vs. untreated HT29 cells; °*p* < 0.001 for siPERK(+ doxy)-treated cells and PD98059-treated cells vs. the corresponding cell type in siPERK(−doxy) cells

We next generated HT29/MDR, HT29/Tg, HT29/Tun, HT29/Bfa clones transduced with a doxycycline-inducible shRNA for *PERK*. In parallel, we treated these clones with the MEK/ERK inhibitor PD98059, which prevents the phosphorylation and transcriptional activity of Nrf2 in colon cancer [23]. As expected, the silencing of *PERK* reduced the phosphorylation on serine 51 of eIF2α (Fig. 4a), a typical PERK substrate [13]. Both *PERK*-silenced cells and PD98059-treated cells showed decreased nuclear translocation of Nrf2 (Fig. 4b), *MRP1* mRNA level (Fig. 4c) and MRP1 amount on the cell surface (Fig. 4d; Additional file. 7a, b), coupled with increased doxorubicin accumulation (Fig. 4e).

Direct downstream targets of PERK, selected using GeneOntology database (www.geneontology.org/), such as eukaryotic translation initiation factor 2 subunit 1 (*EIF2S1*), activating transcription factor 4 (*ATF4*)*,* and *Nrf2* (13, 14; Additional file 8a), were up-regulated in HT29/MDR and HT29/Tun cells (Additional file 8b), in accord with the increased amount of PERK in these cells. *PERK*-silencing significantly reduced the expression levels of all these genes (Additional file 8b). Other upstream controllers of Nrf2 such as glycogen synthase kinase 3β (*GSK3β*)*,* c-Jun N-terminal kinase 1 (*JNK1*), mitogen activated kinase 1 (*MAPK1*)*,* phosphatidylinositol-4,5-bisphosphate 3-kinase catalytic subunit δ (*PI3KCD*), protein kinase Cα (*PRKCA*)*,* were increased in HT29/MDR and HT29/Tun cells, but variably modulated by *PERK*-silencing, likely because multiple pathways control the transcription of these genes. We also analyzed the expression of Nrf2-target genes (www.geneontology.org/), divided into two main categories: 1) genes encoding for anti-oxidant/detoxifying enzymes and chaperones, such as glutathione-disulfide reductase (*GSR*), glucose-6-phosphate dehydrogenase (*G6PD*), thioredoxin reductase 1 *(TXNRD1*), superoxide dismutase 1 (*SOD1*), heme oxygenase 1 (*HMOX1*), NAD(P)H quinone dehydrogenase 1 (*NQO1*)*,* stress induced phosphoprotein 1 (*STIP1*); 2) genes encoding for membrane efflux transporters like *MRP1* (Additional file 8a). All these genes were significantly up-regulated in HT29/MDR cells and HT29/Tun cells and significantly down-regulated in both populations by either *PERK*-silencing or Nrf2-inhbition (Additional file 8b).

**Fig. 5:**
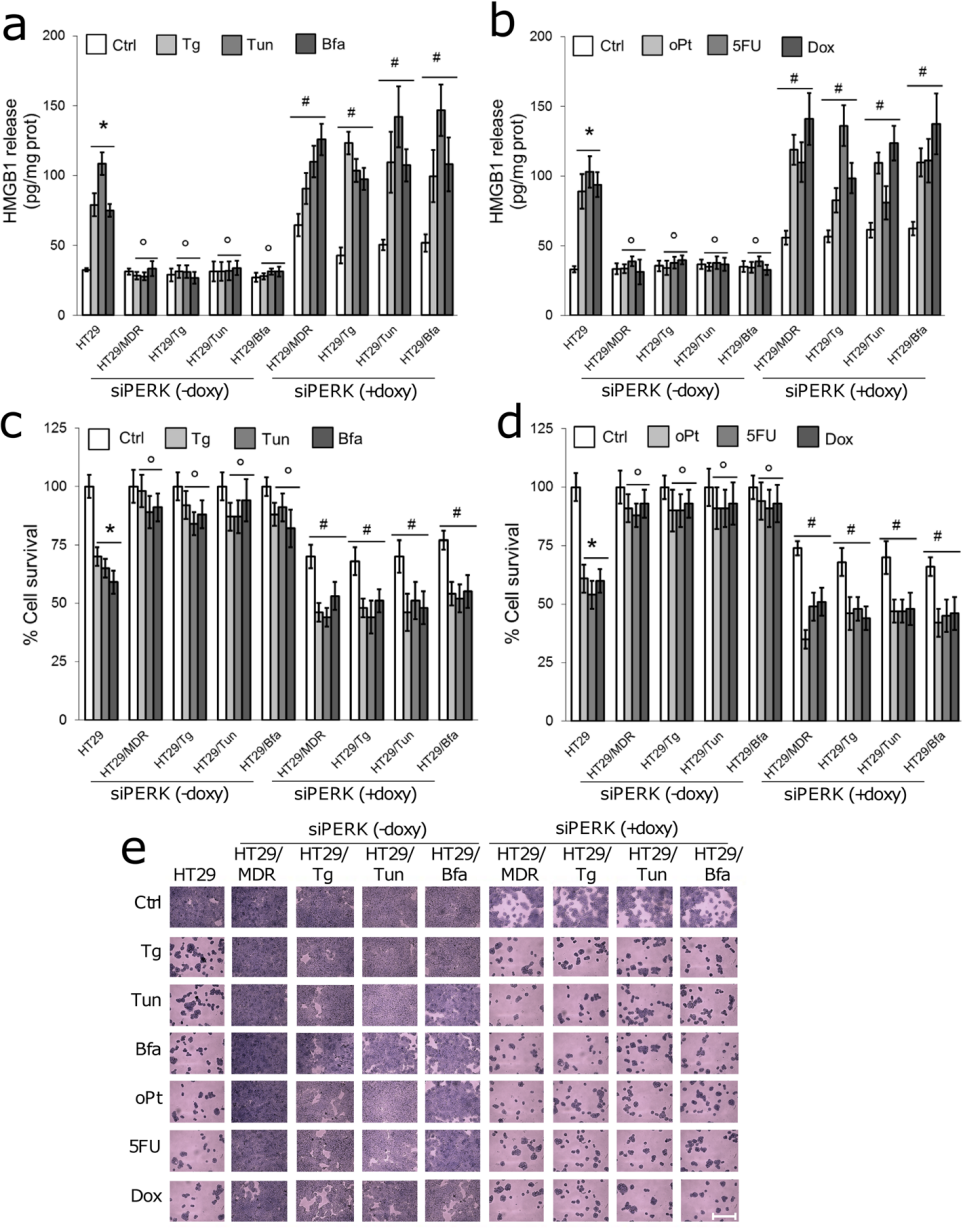
Role of the PERK/Nrf2 axis in resistance to chemotherapy and to ER stress. Human chemoresistant colon cancer HT29/MDR cells and ER stress-resistant clones (HT29/Tg, HT29/Tun, HT29/Bfa) were stably and inducibly silenced for *PERK* (siPERK). Silencing was induced by doxycycline (doxy, 1 μg/mL, 72 h). HT29 cells were used as control of chemosensitive/ER stress-sensitive cells. **a**, **b**. Release of the necrosis marker HMGB1 to culture media of the indicated cells following incubation in fresh medium (Ctrl), or in media containing: thapsigargin (Tg), tunicamycin (Tun), brefeldin A (Bfa), oxaliplatin (oPt), 5-fluorouracil (5FU), doxorubicin (Dox), as indicated in Methods. Data are mean ± SD (*n* = 3). *p < 0.001 for treated cells vs. Ctrl HT29 cells; °p < 0.001 for siPERK – doxy cells vs. HT29 cells treated with the same agent; ^#^p < 0.05 for siPERK + doxy cells vs. siPERK – doxy cells treated with the same agent. **c**, **d**. Viability of cells measured by Neutral red staining. Data are mean ± SD (*n* = 4). *p < 0.001 for treated cells vs. Ctrl HT29 cells; °*p* < 0.01 for siPERK – doxy cells vs. HT29 cells treated with the same agent; ^#^ *p* < 0.001 for siPERK + doxy cells vs. siPERK – doxy cells treated with the same agent. **e**. Crystal violet staining of cells grown in 96-well plates and treated as in **a** and **b**. The images are representatives of at least 5 microscopic fields showing similar cell density. Bar = 500 μM

The inducible silencing of *PERK* increased the release of HMGB1 (Fig. 5a, b) and reduced cell survival (Fig. 5c-e). ER stress inducers and chemotherapeutic drugs enhanced the effect of *PERK* silencing in all the resistant clones (Fig 5). Similar effects were observed upon inhibition of Nrf2 (Additional file 9a-d).

**Fig. 6:**
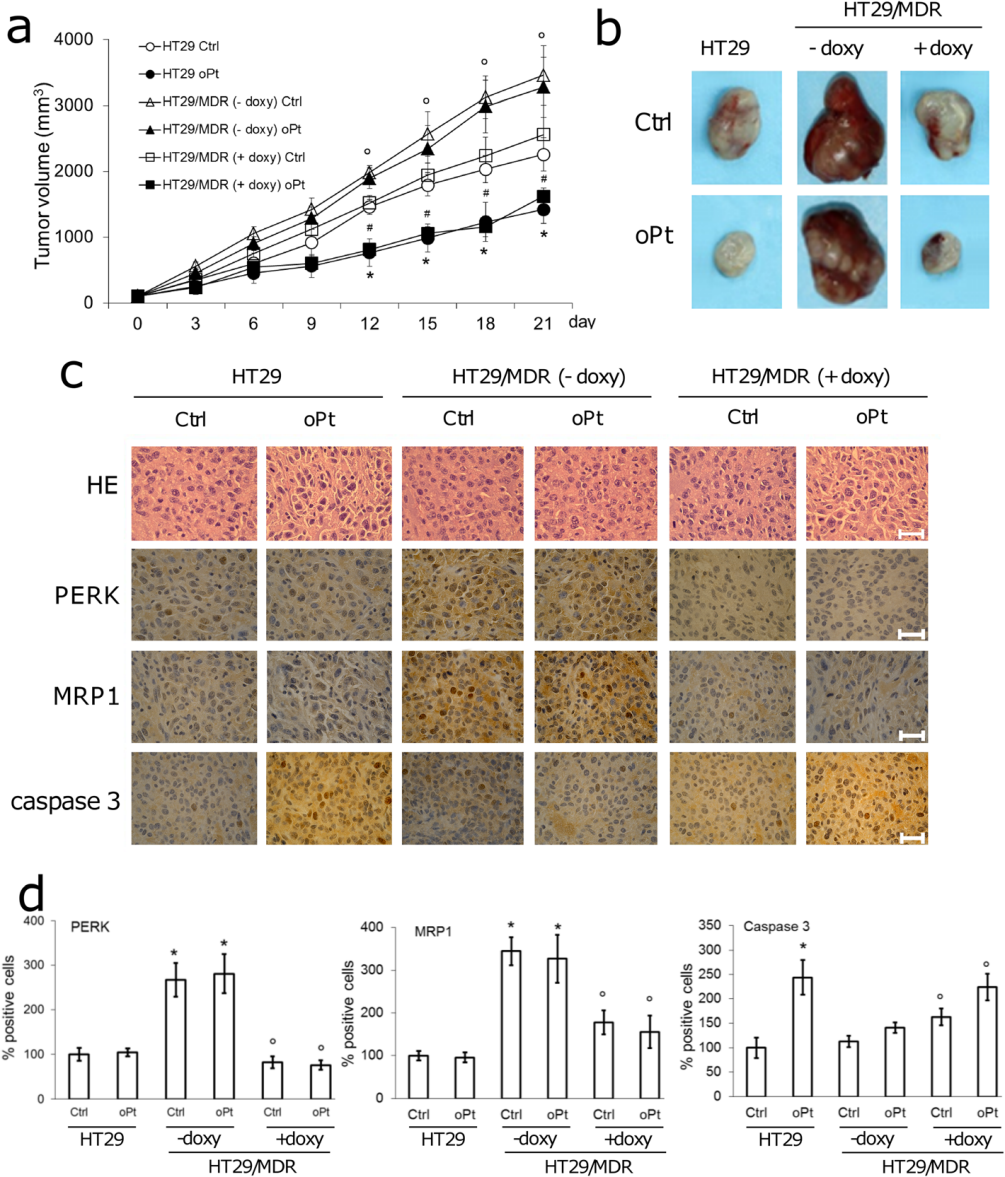
The role of PERK in chemosensitivity in vivo. **a**. Tumor growth of HT29 and HT29/MDR, inducibly silenced for *PERK*, untreated (Ctrl) or treated with oxaliplatin (oPt), as indicated in Methods. Data are mean ± SD (15 mice/group). *p < 0.01 for HT29 oPt vs. HT29 Ctrl group; °*p* < 0.005 for HT29/MDR Ctrl – doxy vs. HT29 Ctrl, HT29/MDR oPt – doxy vs. HT29 oPt group; ^#^ *p* < 0.005 for HT29/MDR Ctrl + doxy vs. HT29/MDR Ctrl – doxy, HT29/MDR oPt + doxy vs. HT29/MDR oPt – doxy. **b**. Photographs of representative tumors of each group. **c** Sections of tumors from each group of animals stained with hematoxylin and eosin (HE) or with the indicated antibodies. Nuclei were counter-stained with hematoxylin. Bar = 10 μm. The photographs are representative of sections from 5 tumors. **d**. Immunostaining quantification. Percentage of PERK, MRP1 and cleaved caspase 3-positive cells was determined in sections from 5 animals of each group (91–109 cells/field), using Photoshop program. *p < 0.002 for HT29/MDR Ctrl – doxy vs HT29 Ctrl, HT29/MDR oPt – doxy vs HT29 oPt, °*p* < 0.02 for HT29/MDR Ctrl + doxy vs HT29/MDR Ctrl – doxy, HT29/MDR oPt + doxy vs HT29/MDR oPt – doxy

Since the expression of *PERK* and *NRF2* is variably related to patient clinical outcome (Additional file 10), to better clarify the impact of *PERK* on tumor progression and response to therapy in a preclinical model, we implanted HT29/MDR cells transduced with inducible shRNA for *PERK* in NOD SCID BALB/c mice, treated with oxaliplatin, with or without doxycycline. HT29 cells were implanted to establish a control oxaliplatin-sensitive tumor. HT29/MDR cells generated tumors faster than HT29 cells (Fig. 6a; Additional file 11). Although we did not detect significant differences in the proliferation rate between sensitive and resistant clones in vitro [7], HT29/MDR cells displayed higher migration (Additional file 12), indicating a higher aggressive phenotype. This may explain the faster growth of tumors derived from HT29/MDR cells. Oxaliplatin treatment alone reduced tumor growth and increased the percentage of apoptotic cells in the HT29-derived tumors but not in the HT29/MDR tumors, which were strongly positive for PERK and MRP1 (Fig. 6a-d).

Administration of doxycycline decreased HT29/MDR tumor growth (Fig. 6a, b), reduced the percentage of cells positive for PERK and MRP1 and increased the number of cells positive for cleaved caspase 3 (Fig. 6c, d). The antitumor effect of oxaliplatin against HT29/MDR tumors was fully restored in doxycycline-treated animals, showing a significant decrease in tumor growth, in line with that of oxaliplatin-treated HT29 tumors (Fig. 6a, b), a decrease in the expression of PERK and MRP1, an increase in intratumor apoptosis (Fig. 6c, d).

## Discussion

Chemotherapeutic agents act at least in part by triggering ER stress [3, 4]. We have recently demonstrated that cells with an acquired resistance to chemotherapy are also resistant to ER stress-triggered cell death [7]. Here we demonstrated that also the inverse sequence of events occurs in cancer cells: the progressive adaptation of chemosensitive cells to ER stress inducers selects cells that are resistant to multiple chemotherapeutic drugs. The acquisition of this double-resistant phenotype, regardless of the ER stress inducer used or the tumor type, was paralleled by the up-regulation of MRP1.

The correlation between acute exposure to ER stress inducers and MRP1 is matter of debate. For instance, thapsigargin did not change MRP1 level in prostate cancer cells [24], but increased MRP1 in colon and lung cancer [25]. Acute exposure to tunicamycin alters the glycosylation of MRP1 and MRP4 and increases the resistance to oxaliplatin in ovarian cancer cells, but under these conditions MRP4 is apparently the prominent player in chemoresistance [26]. Acute exposure to brefeldin A prevents MRP1 translocation from Golgi apparatus to plasma-membrane [27]. Our study differs from the previous ones because it was aimed at selecting clones adapted to survive in prolonged conditions of mild ER stress. This approach simulates the process that occurs in solid tumors constantly facing several conditions inducing ER stress, such as nutrient deprivation, hypoxia, radiotherapy or chemotherapy [1–4].

By comparing of the expression of UPR-related genes we noticed that PERK was the only gene significantly up-regulated in both cells selected for chemoresistance and cells selected for ER stress resistance. This analysis led us to investigate whether the activation of PERK was responsible for the resistance to either ER stress or chemotherapy.

Depending on tumor type and activation kinetics, PERK may promote cell death or survival [28, 29]. Such pleiotropism is reflected by the highly variable and tumor-dependent prognostic role of PERK and Nrf2 in human tumors: mutations in specific oncogenes or oncosuppressor genes, factors related to the tumor micro-environment and immune-system anti-tumor activity, different treatments used in patients likely explain the great variability linking *PERK/Nrf2* expression and clinical outcome.

Contrasting findings have been reported also on the role of PERK in response to anti-cancer treatments. For instance, acute activation of PERK is required for colon cancer cell death mediated by sulindac [30], mesalamine derivatives [31] and histone deacetylase inhibitors [32], suggesting a chemosensitizing role for PERK. In contrast, the prolonged activation of PERK and consequent phosphorylation of EIF2α counteract the cytotoxic effects of tumor necrosis factor-α (TNF-α) and bortezomib [33], docosahexaenoic acid and TNF-related apoptosis inducing ligand [34], thereby preventing the UPR-mediated cell death. These findings are in line with our observations: indeed, clones exhibiting constitutively active PERK were more resistant to cell death triggered either by ER stress or by chemotherapy. The constant challenge with ER stress inducers selected clones with a constitutive up-regulation of PERK and MRP1. Our finding that the PERK-induced Nrf2 activation up-regulates MRP1 expression provides the mechanism by which ER stress induces MRP1 and subsequent MRP1-dependent chemoresistance.

The correlation between Nrf2 and chemoresistance is well known, in particular in colon cancer, where Nrf2 even serves as a marker of chemoresistance [35, 36]. The resistance to platinum-derivatives in Nrf2 expressing cells is usually attributed to the simultaneous up-regulation of antioxidant enzymes, phase II metabolizing enzymes and drug efflux transporters, including MRP1 [37, 38]. Both antioxidant enzymes and MRP1 were indeed up-regulated in ER stress-resistant cancer cells, explaining the refractoriness of these clones to oxaliplatin and cisplatin. The strong up-regulation of MRP1 may explain the simultaneous resistance of ER stress-resistant clones to 5-fluorouracil and doxorubicin, two other substrates of MRP1.

Of note, we also detected a similar activation of PERK/Nrf2/MRP1 axis in the HT29/MDR clone, i.e. a clone with an acquired resistance to chemotherapy and ER stress [7]. It is known that stepwise selection with doxorubicin led to increased Nrf2 expression in ovary cancer cells [39]. It is likely that the same process occurred in HT29/MDR cells, selected with increasing concentration of doxorubicin [20]. Indeed, the analysis of upstream and downstream targets of PERK and Nrf2 in *PERK*-silenced and Nrf2-inhibited cells revealed a strong parallelism between chemoresistant and ER stress-resistant cells: in both populations PERK and its downstream targets are up-regulated, and PERK controls Nrf2 expression. The PERK/Nrf2 axis in turn finely controls the expression of specific gene sets, involved in the protection from oxidant and xenobiotic agents like chemotherapeutic drugs. We thus propose that PERK/Nrf2-controlled genes, including MRP1, may be critical for the acquisition and maintenance of the dual resistance to chemotherapy and ER stress.

Disrupting PERK/Nrf2 axis re-sensitized both ER stress-resistant clones and chemoresistant clones to ER stress-triggered and chemotherapy-triggered cell death, overcoming the double- and cross-resistant phenotype. In preclinical models of colon cancer, PERK-inhibition made ER stress-resistant/MDR tumors as responsive to oxaliplatin as ER stress-sensitive/chemosensitive tumors: this chemosensitizing effects was likely due to the decreased expression of MRP1 that restored the pro-apoptotic effects of oxaliplatin in resistant tumors.

## Conclusions

We demonstrated for the first time that adaptation to ER stress leads to the acquisition of a MDR phenotype in different tumor types, as a consequence of the constitutive activation of PERK/Nrf2/MRP1 axis. Disrupting this axis may overcome MRP1-dependent chemoresistance, opening new perspectives for the treatment of aggressive and resistant solid tumors.

## Additional files

Additional file 1: IC_50_ (μM) of chemotherapeutic agents and ER stress inducers in HT29 and HT29/MDR cells. Data are mean ± SD (*n* = 3). *p < 0.02 for HT29/MDR vs. HT29 cells. (XLSX 12 kb)

Additional file 2: Primers used in qRT-PCR experiments. (XLSX 12 kb)

Additional file 3: Effect of oxaliplatin in 3D-cultures of colon cancer cells. Human chemosensitive HT29 cells, chemoresistant HT29/MDR cells and ER stress-resistant HT29/Tun cells were cultured 7 days embedded in Biomimesys™ matrix to generate 3D-systems. Medium was replaced with fresh medium (Ctrl) or with medium containing 10 μM oxaliplatin (oPt) for 48 h, then cells were analysed by contrast phase microscope. The images are representative of 3 independent experiments. Bar = 50 μM. (TIF 1680 kb)

Additional file 4: Effects of chemotherapeutic drugs in human chemosensitive breast cancer cells with acquired resistance to ER stress. **a**. Release of the necrosis marker HMGB1 to culture media of human chemosensitive breast cancer MCF7 cells and the ER stress-resistant clone MCF7/Tun, grown in fresh medium (Ctrl) or in media containing: thapsigargin (Tg), tunicamycin (Tun), brefeldin A (Bfa), doxorubicin (Dox), cisplatin (Pt), as indicated in Methods. Data are mean ± SD (*n* = 3). *p < 0.001 vs MCF7 Ctrl cells; °*p* < 0.001 for MCF7/Tun treated cells vs MCF7 treated cells. **b**. Viability of cells measured by Neutral red staining. Data are mean ± SD (*n* = 3). *p < 0.05 vs MCF7 Ctrl cells; °*p* < 0.02 for MCF7/Tun treated cells vs MCF7 treated cells. **c**. Whole cell lysates were analyzed for the expression of MRP1. β-tubulin expression was used as control of equal protein loading. The figure is representative of 3 experiments with similar results. **d**. *MRP1* mRNA levels were measured by qRT-PCR. Data are mean ± SD (*n* = 3). *p < 0.01 vs MCF7 cells. **e**. MRP1 protein on cell surface was measured by flow cytometry. *Left panel*: data are presented as mean fluorescence intensity (MFI) ± SD (*n* = 3). *p < 0.02 vs MCF7 cells. *Right panel*: representative flow cytometry histograms. Grey peak: non immune isotypic antibody. **f.** Intracellular doxorubicin content, an index of MRP1 activity, measured by fluorimetry. Data are mean ± SD (*n* = 3). *p < 0.005 vs MCF7 cells. (TIF 1434 kb)

Additional file 5: Effects of chemotherapeutic drugs in human chemosensitive osteosarcoma cells with acquired resistance to ER stress. **a**. Release of the necrosis marker HMGB1 to culture media of human chemosensitive osteosarcoma U-2OS cells and the ER stress-resistant clone U-2OS /Tun, grown in fresh medium (Ctrl) or in media containing: thapsigargin (Tg), tunicamycin (Tun), brefeldin A (Bfa), doxorubicin (Dox), cisplatin (Pt), as indicated in Methods. Data are mean ± SD (*n* = 3). *p < 0.005 vs U-2OS Ctrl cells; °*p* < 0.005 for U-2OS/Tun treated cells vs U-2OS treated cells. **b**. Viability of cells measured by Neutral red staining. Data are mean ± SD (*n* = 3). *p < 0.02 vs U-2OS Ctrl cells; °*p* < 0.05 for U-2OS/Tun treated cells vs U-2OS treated cells. **c**. Whole cell lysates were analyzed for the expression of MRP1. β-tubulin expression was used as control of equal protein loading. The figure is representative of 3 experiments with similar results. **d**. *MRP1* mRNA levels were measured by qRT-PCR. Data are mean ± SD (*n* = 3). *p < 0.005 vs U-2OS cells. **e**. MRP1 protein on cell surface was measured by flow cytometry. *Left panel*: data are presented as mean fluorescence intensity (MFI) ± SD (*n* = 3). *p < 0.02 vs U-2OS cells. *Right panel*: representative flow cytometry histograms. Grey peak: non immune isotypic antibody. **f.** Intracellular doxorubicin content, an index of MRP1 activity, measured by fluorimetry. Data are mean ± SD (*n* = 3). *p < 0.05 vs U-2OS cells. (TIF 1533 kb)

Additional file 6: Expression of genes involved in the UPR in HT29, HT29/Tun and HT29/MDR cells. ERAD: ER-associated degradation; UPR: unfolded protein response; ERQC: ER-quality control. Fold-Change (2^(− Delta Delta Ct)) is the normalized gene expression (2^(− Delta Ct)) in HT29/Tun or HT29/MDR cells, divided the normalized gene expression (2^(− Delta Ct)) in HT29 cells (*n* = 4), where Ct is the threshold cycle in qRT-PCR. Fold-change values greater than 1 indicate up-regulation, fold-change values less than 1 indicate down-regulation. The p values are calculated based on a Student’s *t*-test of the replicate 2^(− Delta Ct) values for each gene. *p* < 0.05 was considered significant. Genes significantly up- or down-regulated more than 2-fold either in HT29/Tun or HT29/MDR cells are in bold characters. (XLSX 16 kb)

Additional file 7: MRP1 expression on cell surface of sensitive and resistant colon cancer cells upon PERK/Nrf2 inhibition. **a**, **b**. Representative flow cytometry histograms of MRP1 protein in human chemoresistant colon cancer HT29/MDR cells and ER stress-resistant clones (HT29/Tg, HT29/Tun, HT29/Bfa), stably and inducibly transduced with a silencing vector for *PERK*, or treated with PD98059 (10 μM, 72 h), which blocks Nrf2 nuclear translocation. HT29 were included as control of chemosensitive/ER stress-sensitive cells. Grey peaks: non immune isotypic antibody. (TIF 1205 kb)

Additional file 8: Expression of PERK- and Nrf2*-*upstream and downstream genes in chemoresistant and ER stress-resistant cells. **a**. Schematic representation of upstream and downstream targets of PERK and Nrf2. Blue box: genes upstream PERK; red boxes: genes downstream PERK; orange boxes: genes upstream Nrf2; green boxes: genes downstream Nrf2. **b**. Relative expression, indicated in a colorimetric scale, of the indicated PERK- and Nrf2-upstream and downstream genes, in HT29/MDR and HT29/Tun cells, grown un fresh medium (Ctrl), transduced with a silencing vector for *PERK*, or treated with PD98059 (PD; 10 μM, 72 h), measured by RT-PCR. The expression of each gene in HT29 cells, used as internal control, was considered equal to 1. Data are mean ± SD (*n* = 4). *p < 0.05 for untreated (Ctrl) HT29/MDR or HT29/Tun cells vs. HT29 cells; °*p* < 0.05 for significantly reduced genes in *siPERK*- and PD-treated cells vs. respective untreated (Ctrl) HT29/MDR or HT29/Tun cells. *GRP78*: glucose-regulated protein 78; *EIF2S1*: eukaryotic translation initiation factor 2 subunit 1; *ATF4*: activating transcription factor 4; *GSK3β*: glycogen synthase kinase 3β; *JNK1*: c-Jun N-terminal kinase 1; *MAPK1*: mitogen activated kinase 1; *PI3KCD*: phosphatidylinositol-4,5-bisphosphate 3-kinase catalytic subunit δ; *PRKCA*: protein kinase Cα; *NFKB*: nuclear factor-kB; *GSR*: glutathione-disulfide reductase; *G6PD*: glucose-6-phosphate dehydrogenase; *TXNRD*1: thioredoxin reductase 1; *SOD1*: superoxide dismutase 1; *HMOX1*: heme oxygenase 1; *NQO*1: NAD(P)H quinone dehydrogenase 1; *STIP1*: stress induced phosphoprotein 1. (TIF 836 kb)

Additional file 9: Nrf2 inhibition reverses the resistance to chemotherapy and ER stress. Human chemoresistant colon cancer HT29/MDR cells and ER stress-resistant clones (HT29/Tg, HT29/Tun, HT29/Bfa) were grown in the absence or in the presence of PD98059 (10 μM, 72 h), which blocks Nrf2 nuclear translocation. **a**, **b**. Release of the necrosis marker HMGB1 to culture media of the indicated cells following incubation in fresh medium (Ctrl), or in media containing: thapsigargin (Tg), tunicamycin (Tun), brefeldin A (Bfa), oxaliplatin (oPt), 5-fluorouracil (5FU), doxorubicin (Dox), as indicated in Methods. Data are mean ± SD (*n* = 3). *p < 0.001 vs HT29 Ctrl cells; °*p* < 0.001 for HT29/MDR, HT29/Tg, HT29/Tun, HT29/Bfa vs HT29 cells; ^#^ *p* < 0.001 for PD98059-treated cells vs PD98059-untreated cells. **c**, **d**. Viability of cells measured by Neutral red staining. *p < 0.005 vs HT29 Ctrl cells; °*p* < 0.01 for HT29/MDR, HT29/Tg, HT29/Tun, HT29/Bfa vs HT29 cells; ^#^ *p* < 0.02 for PD98059-treated cells vs PD98059-untreated cells. (TIF 3494 kb)

Additional file 10: Correlation between *PERK* or *NRF*2 expression and patient clinical outcome in different tumors. Patient overall survival was calculated by Cox's proportional hazard model and Kaplan-Meier method using the GSEA software. Z score: correlation score between gene expression and survival. FDR: false discovery rate. ACC: adrenocortical carcinoma; BRCA: breast invasive carcinoma; BLCA: bladder urothelial carcinoma; CESC: cervical squamous cell carcinoma and endocervical adenocarcinoma; CHOL: cholangiocarcinoma; COAD: colon adenocarcinoma; COADREAD: colorectal adenocarcinoma; DLBC: diffuse large B-cell lymphoma; GBM: glioblastoma multiforme; HNSC: head and neck squamous cell carcinoma; KICH: chromophobe renal cell carcinoma; KIRC: kidney renal clear cell carcinoma; KIRP: kidney renal papillary cell carcinoma; LGG: lower grade glioma; LIHC: liver hepatocellular carcinoma; LUAD: lung adenocarcinoma; LUSC: lung squamous cell carcinoma; MESO: mesothelioma; OV: ovarian serous cystadenocarcinoma; PAAD: pancreatic adenocarcinoma; PCPG: pheochromocytoma and paraganglioma; PRAD: prostate adenocarcinoma; READ: rectum adenocarcinoma; SARC: sarcoma; SKCM: skin cutaneous melanoma; TGCT: testicular germ cell tumor; THCA: thyroid carcinoma; THYM: thymoma; UCEC: uterine corpus endometrial carcinoma; UCS: uterine carcinosacoma; UVM: uveal melanoma. Significant p values are in bold characters. (XLSX 17 kb)

Additional file 11: Tumor growth in mice bearing HT29 and HT29/MDR tumors. Tumor volume (reported in mm^3^) of HT29 and HT29/MDR, inducibly silenced for PERK, untreated (Ctrl) or treated with oxaliplatin (oPt), as indicated in Methods and in the main Figure 6, at different time points for each animal. (XLSX 24 kb)

Additional file 12: Migration of sensitive and resistant colon cancer cells. Migration ability of HT29, HT29/MDR and HT29/Tun cells was evaluated as capacity to close the wound over a period of 24 h. **a**. Representative images of 1 out of 3 experiments. Bar: 200 μm. **b**. Quantification of migration rate. Data are mean ± SD (*n* = 3). *p < 0.002 vs HT29 cells; °*p* < 0.001 vs HT29/MDR cells. (TIF 2275 kb)

## Abbreviations

ANOVA: Analysis of variance
ATF4: Activating transcription factor 4
ATF6: activating transcription factor 6
C/EBP-β LIP: CAAT/enhancer-β liver-enriched inhibitory protein
ChIP: Chromatin immunoprecipitation
CHOP: C/EBP homologous protein
EIF2AK3: Eukaryotic translation initiation factor-2α kinase 3
EIF2S1: Eukaryotic translation initiation factor 2 subunit 1
ER: Endoplasmic reticulum
ERAD: ER-associated degradation
FBS: Foetal bovine serum
G6PD: Glucose-6-phosphate dehydrogenase
GRP78: Glucose-regulated protein 78
GSK3β: Glycogen synthase kinase 3β
GSR: Glutathione-disulfide reductase
HMGB1: High Mobility Group Protein 1
HMOX1: Heme oxygenase 1
IRE1: Inositol-requiring enzyme 1
JNK1: c-Jun N-terminal kinase 1
MAPK1: Mitogen activated kinase 1
MDR: Multidrug resistance
MRP1, 2, 3, 4: MDR related protein 1, 2, 3, 4
NQO1: NAD(P)H quinone dehydrogenase 1
Nrf2: Erythroid-derived 2-like 2
PERK: Protein kinase RNA-like endoplasmic reticulum kinase
Pgp: P-glycoprotein
PI3KCD: Phosphatidylinositol-4,5-bisphosphate 3-kinase catalytic subunit δ
PRKCA: Protein kinase Cα
qRT-PCR: Quantitative real-time polymerase chain reaction
RIPA: Radioimmunoprecipitation assay
shRNA: Short hairpin RNA
SOD1: Superoxide dismutase 1
STIP1: Stress induced phosphoprotein 1
TNF-α: Tumor necrosis factor-α
TRB3: Tribbles-related protein 3
TXNRD1: Thioredoxin reductase 1
UPR: Unfolded protein response
