# Supplementary figures and images for: PERK induces resistance to cell death elicited by endoplasmic reticulum stress and chemotherapy
Source: Mol Cancer. 2017 May 12;16:91. doi: 10.1186/s12943-017-0657-0 (PMC5427528; doi:10.1186/s12943-017-0657-0)

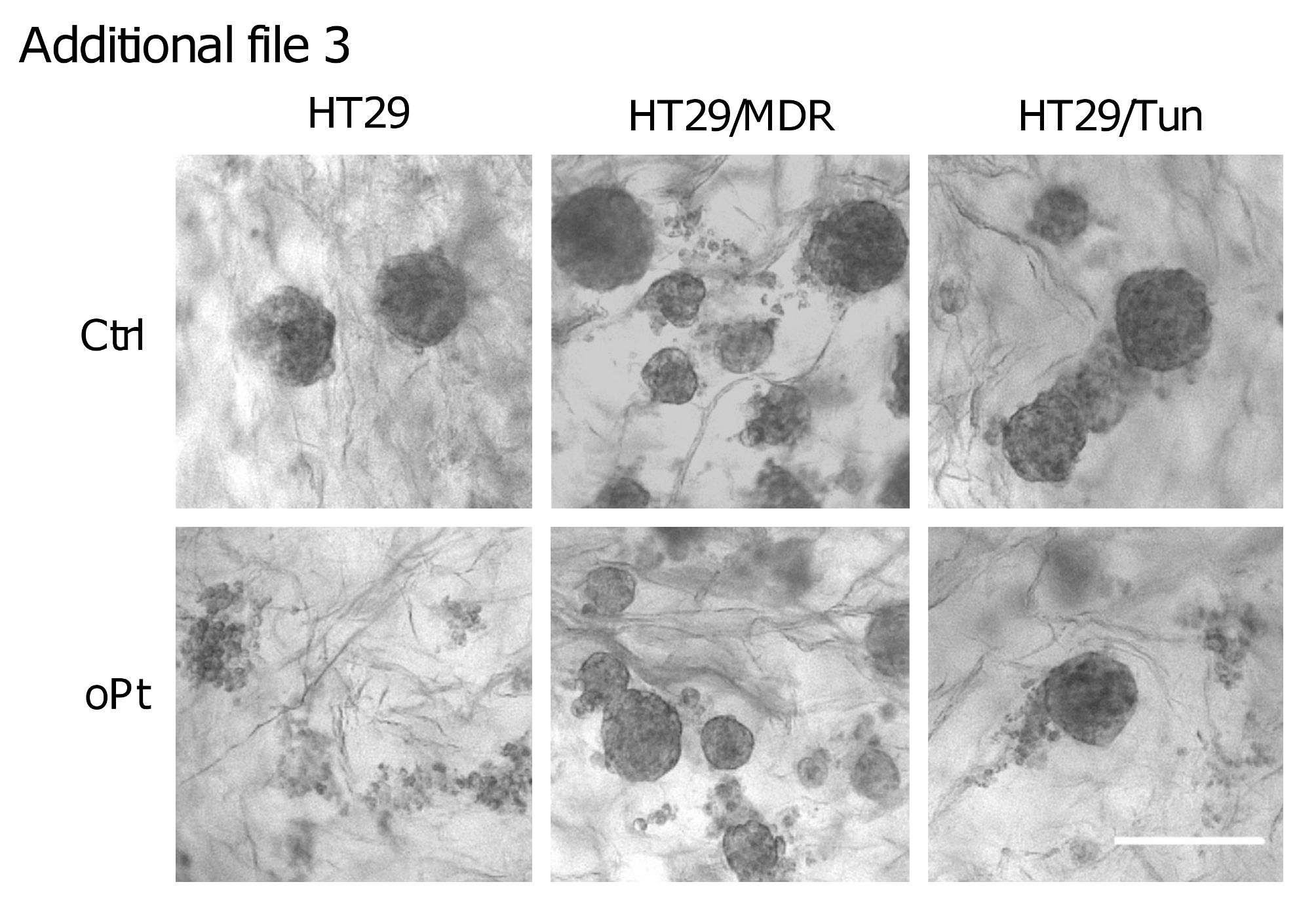

Supplement: Supplementary file 3 — Effect of oxaliplatin in 3D-cultures of colon cancer cells. Human chemosensitive HT29 cells, chemoresistant HT29/MDR cells and ER stress-resistant HT29/Tun cells were cultured 7 days embedded in Biomimesys™ matrix to generate 3D-systems. Medium was replaced with fresh medium (Ctrl) or with medium containing 10 μM oxaliplatin (oPt) for 48 h, then cells were analysed by contrast phase microscope. The images are representative of 3 independent experiments. Bar = 50 μM. (TIF 1680 kb) [file 12943_2017_657_MOESM3_ESM.tif]

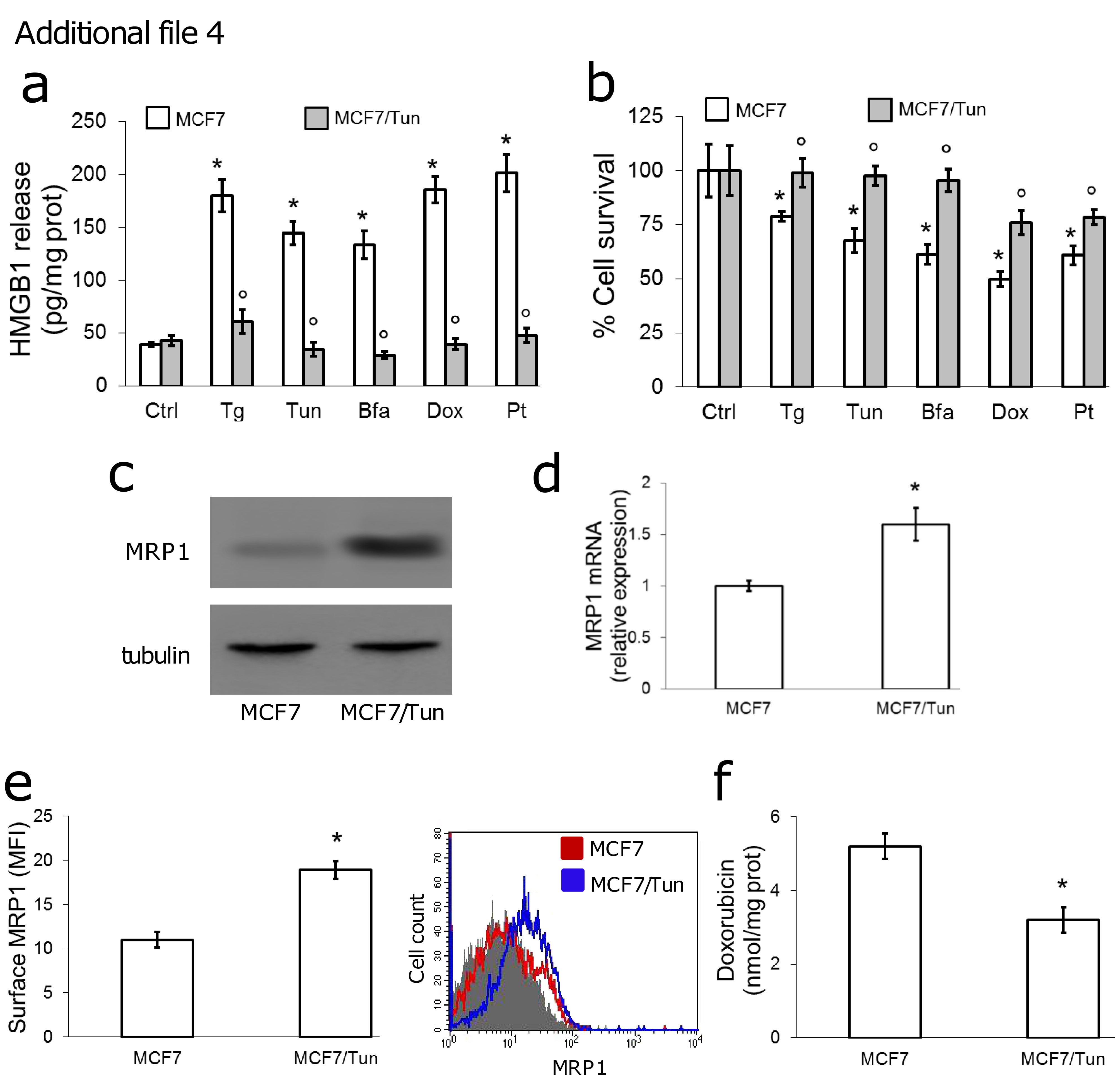

Supplement: Supplementary file 4 — Effects of chemotherapeutic drugs in human chemosensitive breast cancer cells with acquired resistance to ER stress. a. Release of the necrosis marker HMGB1 to culture media of human chemosensitive breast cancer MCF7 cells and the ER stress-resistant clone MCF7/Tun, grown in fresh medium (Ctrl) or in media containing: thapsigargin (Tg), tunicamycin (Tun), brefeldin A (Bfa), doxorubicin (Dox), cisplatin (Pt), as indicated in Methods. Data are mean ± SD (n = 3). *p < 0.001 vs MCF7 Ctrl cells; °p < 0.001 for MCF7/Tun treated cells vs MCF7 treated cells. b. Viability of cells measured by Neutral red staining. Data are mean ± SD (n = 3). *p < 0.05 vs MCF7 Ctrl cells; °p < 0.02 for MCF7/Tun treated cells vs MCF7 treated cells. c. Whole cell lysates were analyzed for the expression of MRP1. β-tubulin expression was used as control of equal protein loading. The figure is representative of 3 experiments with similar results. d. MRP1 mRNA levels were measured by qRT-PCR. Data are mean ± SD (n = 3). *p < 0.01 vs MCF7 cells. e. MRP1 protein on cell surface was measured by flow cytometry. Left panel: data are presented as mean fluorescence intensity (MFI) ± SD (n = 3). *p < 0.02 vs MCF7 cells. Right panel: representative flow cytometry histograms. Grey peak: non immune isotypic antibody. f. Intracellular doxorubicin content, an index of MRP1 activity, measured by fluorimetry. Data are mean ± SD (n = 3). *p < 0.005 vs MCF7 cells. (TIF 1434 kb) [file 12943_2017_657_MOESM4_ESM.tif]

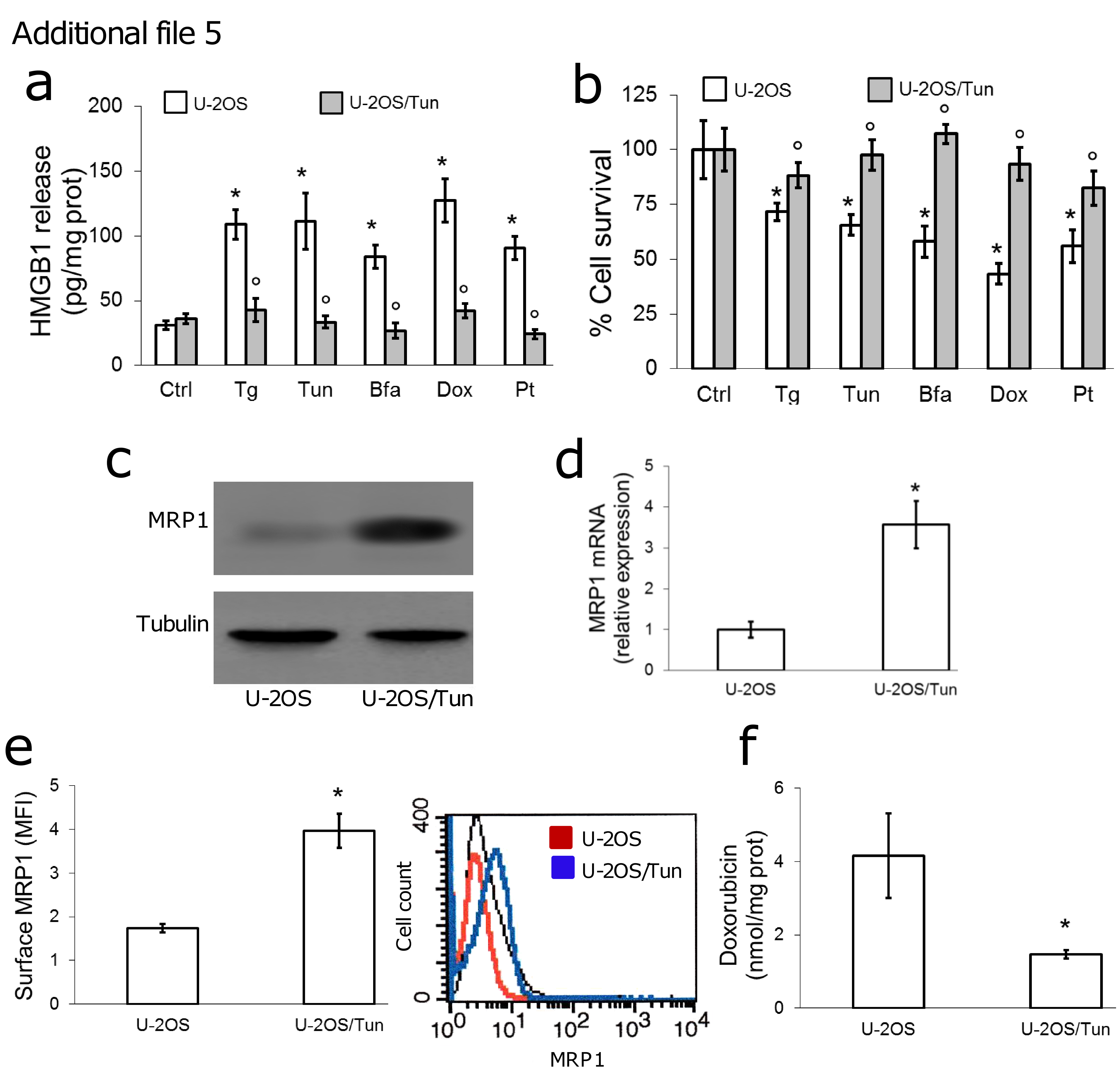

Supplement: Supplementary file 5 — Effects of chemotherapeutic drugs in human chemosensitive osteosarcoma cells with acquired resistance to ER stress. a. Release of the necrosis marker HMGB1 to culture media of human chemosensitive osteosarcoma U-2OS cells and the ER stress-resistant clone U-2OS /Tun, grown in fresh medium (Ctrl) or in media containing: thapsigargin (Tg), tunicamycin (Tun), brefeldin A (Bfa), doxorubicin (Dox), cisplatin (Pt), as indicated in Methods. Data are mean ± SD (n = 3). *p < 0.005 vs U-2OS Ctrl cells; °p < 0.005 for U-2OS/Tun treated cells vs U-2OS treated cells. b. Viability of cells measured by Neutral red staining. Data are mean ± SD (n = 3). *p < 0.02 vs U-2OS Ctrl cells; °p < 0.05 for U-2OS/Tun treated cells vs U-2OS treated cells. c. Whole cell lysates were analyzed for the expression of MRP1. β-tubulin expression was used as control of equal protein loading. The figure is representative of 3 experiments with similar results. d. MRP1 mRNA levels were measured by qRT-PCR. Data are mean ± SD (n = 3). *p < 0.005 vs U-2OS cells. e. MRP1 protein on cell surface was measured by flow cytometry. Left panel: data are presented as mean fluorescence intensity (MFI) ± SD (n = 3). *p < 0.02 vs U-2OS cells. Right panel: representative flow cytometry histograms. Grey peak: non immune isotypic antibody. f. Intracellular doxorubicin content, an index of MRP1 activity, measured by fluorimetry. Data are mean ± SD (n = 3). *p < 0.05 vs U-2OS cells. (TIF 1533 kb) [file 12943_2017_657_MOESM5_ESM.tif]

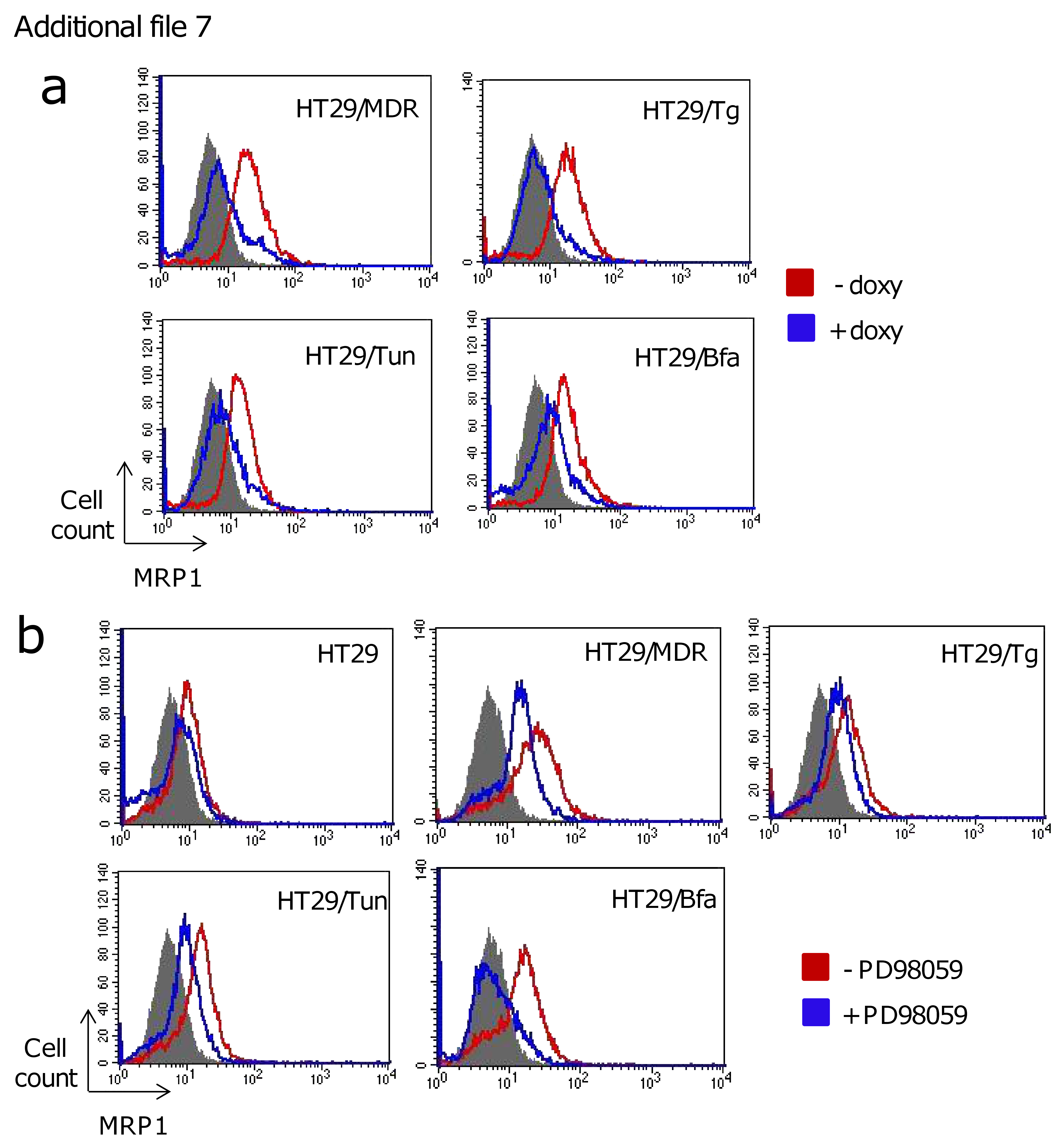

Supplement: Supplementary file 7 — MRP1 expression on cell surface of sensitive and resistant colon cancer cells upon PERK/Nrf2 inhibition. a, b. Representative flow cytometry histograms of MRP1 protein in human chemoresistant colon cancer HT29/MDR cells and ER stress-resistant clones (HT29/Tg, HT29/Tun, HT29/Bfa), stably and inducibly transduced with a silencing vector for PERK, or treated with PD98059 (10 μM, 72 h), which blocks Nrf2 nuclear translocation. HT29 were included as control of chemosensitive/ER stress-sensitive cells. Grey peaks: non immune isotypic antibody. (TIF 1205 kb) [file 12943_2017_657_MOESM7_ESM.tif]

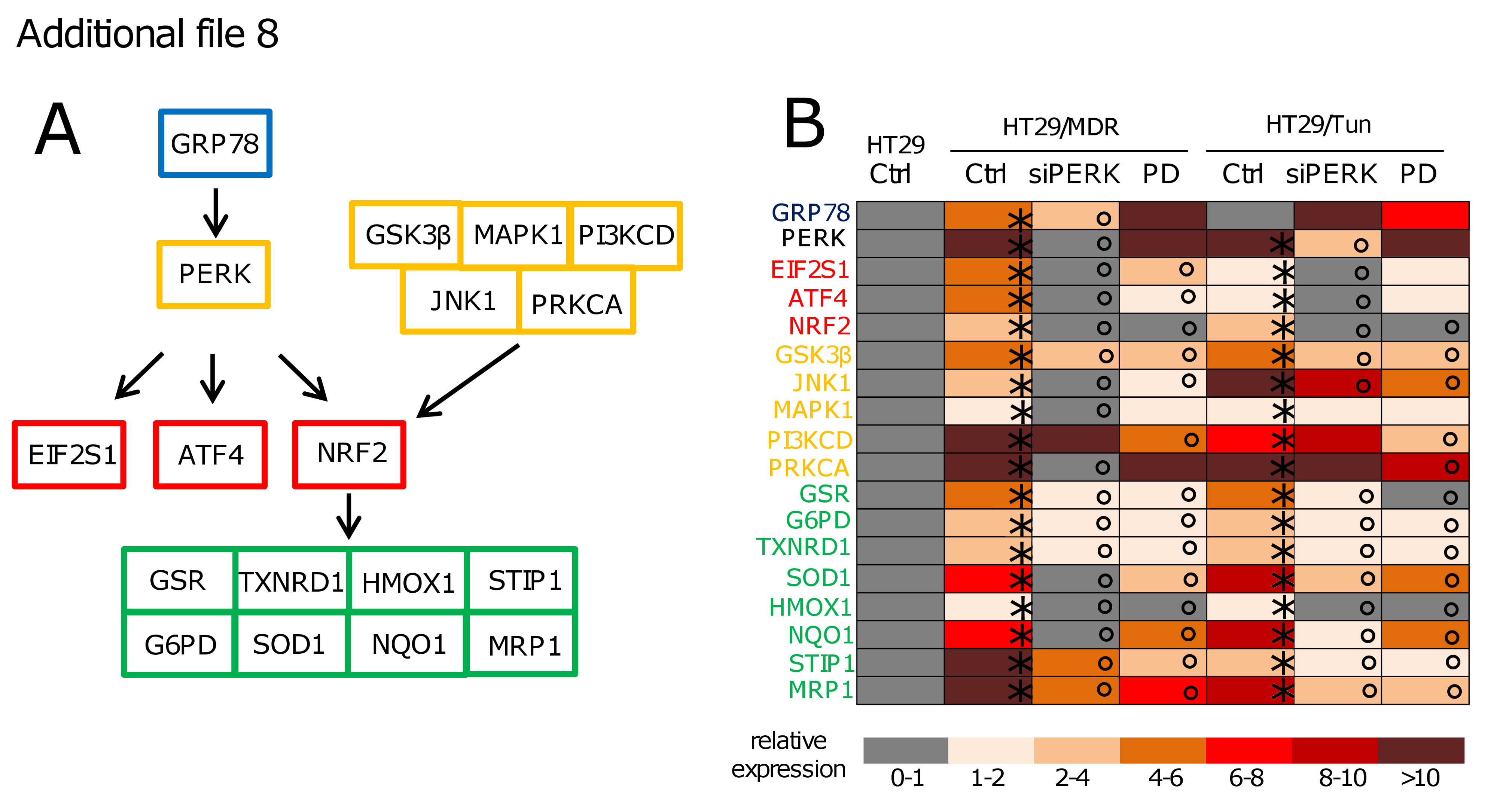

Supplement: Supplementary file 8 — Expression of PERK- and Nrf2-upstream and downstream genes in chemoresistant and ER stress-resistant cells. a. Schematic representation of upstream and downstream targets of PERK and Nrf2. Blue box: genes upstream PERK; red boxes: genes downstream PERK; orange boxes: genes upstream Nrf2; green boxes: genes downstream Nrf2. b. Relative expression, indicated in a colorimetric scale, of the indicated PERK- and Nrf2-upstream and downstream genes, in HT29/MDR and HT29/Tun cells, grown un fresh medium (Ctrl), transduced with a silencing vector for PERK, or treated with PD98059 (PD; 10 μM, 72 h), measured by RT-PCR. The expression of each gene in HT29 cells, used as internal control, was considered equal to 1. Data are mean ± SD (n = 4). *p < 0.05 for untreated (Ctrl) HT29/MDR or HT29/Tun cells vs. HT29 cells; °p < 0.05 for significantly reduced genes in siPERK- and PD-treated cells vs. respective untreated (Ctrl) HT29/MDR or HT29/Tun cells. GRP78: glucose-regulated protein 78; EIF2S1: eukaryotic translation initiation factor 2 subunit 1; ATF4: activating transcription factor 4; GSK3β: glycogen synthase kinase 3β; JNK1: c-Jun N-terminal kinase 1; MAPK1: mitogen activated kinase 1; PI3KCD: phosphatidylinositol-4,5-bisphosphate 3-kinase catalytic subunit δ; PRKCA: protein kinase Cα; NFKB: nuclear factor-kB; GSR: glutathione-disulfide reductase; G6PD: glucose-6-phosphate dehydrogenase; TXNRD1: thioredoxin reductase 1; SOD1: superoxide dismutase 1; HMOX1: heme oxygenase 1; NQO1: NAD(P)H quinone dehydrogenase 1; STIP1: stress induced phosphoprotein 1. (TIF 836 kb) [file 12943_2017_657_MOESM8_ESM.tif]

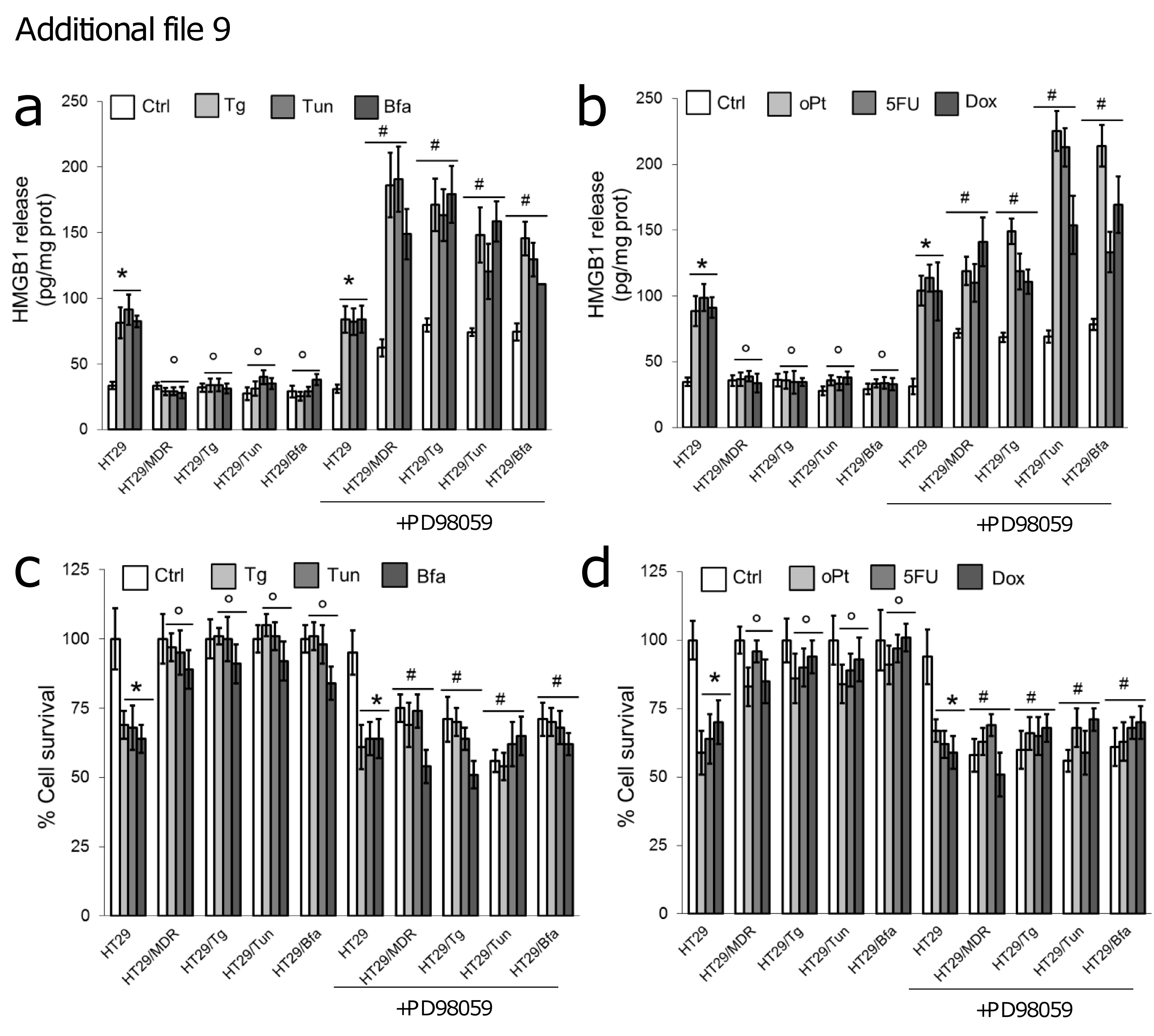

Supplement: Supplementary file 9 — Nrf2 inhibition reverses the resistance to chemotherapy and ER stress. Human chemoresistant colon cancer HT29/MDR cells and ER stress-resistant clones (HT29/Tg, HT29/Tun, HT29/Bfa) were grown in the absence or in the presence of PD98059 (10 μM, 72 h), which blocks Nrf2 nuclear translocation. a, b. Release of the necrosis marker HMGB1 to culture media of the indicated cells following incubation in fresh medium (Ctrl), or in media containing: thapsigargin (Tg), tunicamycin (Tun), brefeldin A (Bfa), oxaliplatin (oPt), 5-fluorouracil (5FU), doxorubicin (Dox), as indicated in Methods. Data are mean ± SD (n = 3). *p < 0.001 vs HT29 Ctrl cells; °p < 0.001 for HT29/MDR, HT29/Tg, HT29/Tun, HT29/Bfa vs HT29 cells; # p < 0.001 for PD98059-treated cells vs PD98059-untreated cells. c, d. Viability of cells measured by Neutral red staining. *p < 0.005 vs HT29 Ctrl cells; °p < 0.01 for HT29/MDR, HT29/Tg, HT29/Tun, HT29/Bfa vs HT29 cells; # p < 0.02 for PD98059-treated cells vs PD98059-untreated cells. (TIF 3494 kb) [file 12943_2017_657_MOESM9_ESM.tif]

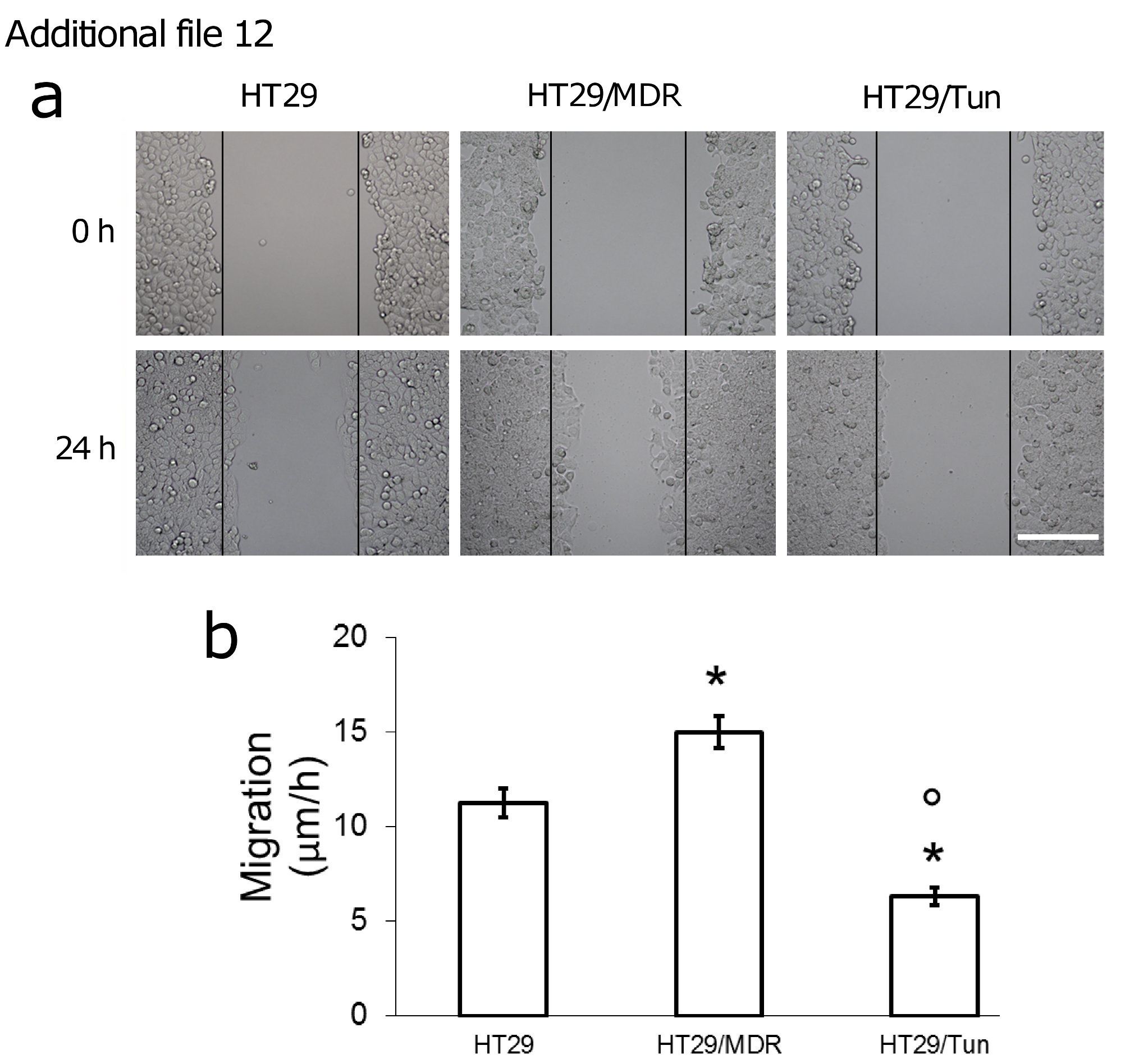

Supplement: Supplementary file 12 — Migration of sensitive and resistant colon cancer cells. Migration ability of HT29, HT29/MDR and HT29/Tun cells was evaluated as capacity to close the wound over a period of 24 h. a. Representative images of 1 out of 3 experiments. Bar: 200 μm. b. Quantification of migration rate. Data are mean ± SD (n = 3). *p < 0.002 vs HT29 cells; °p < 0.001 vs HT29/MDR cells. (TIF 2275 kb) [file 12943_2017_657_MOESM12_ESM.tif]
